# No Reliable Association between Runs of Homozygosity and Schizophrenia in a Well-Powered Replication Study

**DOI:** 10.1371/journal.pgen.1006343

**Published:** 2016-10-28

**Authors:** Emma C. Johnson, Douglas W. Bjelland, Daniel P. Howrigan, Abdel Abdellaoui, Gerome Breen, Anders Borglum, Sven Cichon, Franziska Degenhardt, Andreas J. Forstner, Josef Frank, Giulio Genovese, Stefanie Heilmann-Heimbach, Stefan Herms, Per Hoffman, Wolfgang Maier, Manuel Mattheisen, Derek Morris, Bryan Mowry, Betram Müller-Mhysok, Benjamin Neale, Igor Nenadic, Markus M. Nöthen, Colm O’Dushlaine, Marcella Rietschel, Douglas M. Ruderfer, Dan Rujescu, Thomas G. Schulze, Matthew A. Simonson, Eli Stahl, Jana Strohmaier, Stephanie H. Witt, Schizophrenia Working Group of the Psychiatric Genomics Consortium, Patrick F. Sullivan, Matthew C. Keller

**Affiliations:** 1 Department of Psychology and Neuroscience, University of Colorado at Boulder, United States of America; 2 Institute for Behavioral Genetics, University of Colorado at Boulder, United States of America; 3 Center for Human Genetic Research, Massachusetts General Hospital, Boston, Massachusetts, United States of America; 4 Broad Institute, Cambridge, Massachusetts, United States of America; 5 Analytic and Translational Genetics Unit, Massachusetts General Hospital, Boston, United States of America; 6 Department of Biological Psychology, VU University Amsterdam, Amsterdam, Netherlands; 7 IDepartment of Social Genetic and Developmental Psychiatry, King’s College London, London, United Kingdom; 8 The Lundbeck Foundation Initiative for Integrative Psychiatric Research, iPSYCH, Denmark; 9 Centre for Integrative Sequencing, iSEQ, Aarhus University, Aarhus, Denmark; 10 Department of Biomedicine, Aarhus University, Aarhus, Denmark; 11 Department P, Aarhus University Hospital, Risskov, Denmark; 12 Department of Genomics, Life and Brain Center, University of Bonn, Germany; 13 Division of Medical Genetics, Department of Biomedicine, University Basel, Basel, Switzerland; 14 Institute of Neuroscience and Medicine (INM-1), Structural and Functional Organisation of the Brain, Genomic Imaging, Research Centre Juelich, Juelich, Germany; 15 Institute of Human Genetics, University of Bonn, Bonn, Germany; 16 Department of Genetic Epidemiology in Psychiatry, Central Institute of Mental Health, Medical Faculty Mannheim / Heidelberg University, Mannheim, Germany; 17 Department of Psychiatry, University of Bonn, Bonn, Germany; 18 Department of Psychiatry & Neuropsychiatric Genetics Research Group, School of Medicine, The Trinity Centre for Health Sciences, St. James's Hospital, Ireland; 19 Queensland Centre for Schizophrenia Mental Health Research, The Park, Centre for Mental Health, Wacol, Australia; 20 Department of Psychiatry, University of Queensland, Brisbane, Australia; 21 Max Planck Institute of Psychiatry, Munich, Germany; 22 Munich Cluster for Systems Neurology (SyNergy), Munich, Germany; 23 University of Liverpool, Institute of Translational Medicine, Liverpool, United Kingdom; 24 Department of Psychiatry and Psychotherapy, Jena University Hospital, Jena, Germany; 25 Neuropsychiatric Genetics Research Group, Department of Psychiatry and Institute of Molecular Medicine, Trinity College Dublin, Ireland; 26 Division of Psychiatric Genomics, Department of Psychiatry, Icahn School of Medicine at Mount Sinai, New York, NY, United States of America; 27 Molecular and Clinical Neurobiology, Department of Psychiatry, Ludwig-Maximilians-University, Munich, Germany; 28 Department of Psychiatry, University of Halle-Wittenberg, Halle, Germany; 29 Institute for Psychiatric Phenomics and Genomics (IPPG), Ludwig-Maximilians-University, Munich, Germany; 30 Mayo Clinic, Department of Health Sciences, Division of Biomedical Statistics and Informatics, Rochester, Minnesota, United States of America; 31 Department of Medical Epidemiology and Biostatistics, Karolinska Institute, Stockholm, Sweden; 32 Department of Genetics, University of North Carolina, Chapel Hill, NC, United States of America; 33 Department of Psychiatry, University of North Carolina, Chapel Hill, NC, United States of America; University of Miami, Miller School of Medicine, UNITED STATES

## Abstract

It is well known that inbreeding increases the risk of recessive monogenic diseases, but it is less certain whether it contributes to the etiology of complex diseases such as schizophrenia. One way to estimate the effects of inbreeding is to examine the association between disease diagnosis and genome-wide autozygosity estimated using runs of homozygosity (ROH) in genome-wide single nucleotide polymorphism arrays. Using data for schizophrenia from the Psychiatric Genomics Consortium (n = 21,868), Keller et al. (2012) estimated that the odds of developing schizophrenia increased by approximately 17% for every additional percent of the genome that is autozygous (β = 16.1, CI(β) = [6.93, 25.7], *Z* = 3.44, *p* = 0.0006). Here we describe replication results from 22 independent schizophrenia case-control datasets from the Psychiatric Genomics Consortium (n = 39,830). Using the same ROH calling thresholds and procedures as Keller et al. (2012), we were unable to replicate the significant association between ROH burden and schizophrenia in the independent PGC phase II data, although the effect was in the predicted direction, and the combined (original + replication) dataset yielded an attenuated but significant relationship between *Froh* and schizophrenia (β = 4.86,CI(β) = [0.90,8.83],*Z* = 2.40,*p* = 0.02). Since Keller et al. (2012), several studies reported inconsistent association of ROH burden with complex traits, particularly in case-control data. These conflicting results might suggest that the effects of autozygosity are confounded by various factors, such as socioeconomic status, education, urbanicity, and religiosity, which may be associated with both real inbreeding and the outcome measures of interest.

## Author Summary

It is well known that mating between relatives increases the risk that a child will have a rare recessive genetic disease, but there has also been increasing interest and inconsistent findings on whether inbreeding is a risk factor for common, complex psychiatric disorders such as schizophrenia. The best powered study to date investigating this theory predicted that the odds of developing schizophrenia increase by approximately 17% for every additional percent of the genome that shows evidence of inbreeding. In this replication, we used genome-wide single nucleotide polymorphism data from 18,562 schizophrenia cases and 21,268 controls to quantify the degree to which they were inbred and to test the hypothesis that schizophrenia cases show higher mean levels of inbreeding. Contrary to the original study, we did not find evidence for distant inbreeding to play a role in schizophrenia risk. There are various confounding factors that could explain the discrepancy in results from the original study and our replication, and this should serve as a cautionary note–careful attention should be paid to issues like ascertainment when using the data from genome-wide case-control association studies for secondary analyses for which the data may not have originally been intended.

## Introduction

Close inbreeding (e.g., cousin-cousin mating) is known to decrease fitness in animals[1] and to increase risk for recessive Mendelian diseases in humans[2], a phenomenon known as inbreeding depression. Inbreeding depression is thought to occur due to evolutionary selection against genetic variants that decrease fitness—e.g., variants that increase risk of disorders[3]. Such fitness-reducing variants should not only be more rare, but also more recessive than expected under a neutral evolution model (i.e., show directional dominance). If so, individuals with a greater proportion of their genome in autozygous stretches (two homologous segments of a chromosome inherited from a common ancestor identical by descent [IBD]) should have higher rates of disorders. This is because autozygous regions reveal the full, harmful effects of any deleterious, recessive alleles that existed on the haplotype of the common ancestor.

Whether inbreeding increases risk for complex disorders like schizophrenia is less clear. Previous studies have found that inbreeding is associated with higher rates of complex disorders[4–9]. However, sample sizes have typically been small and the possibility that confounding factors might explain the results has left the links inconclusive. Moreover, close inbreeding accounts for fewer than 1% of marriages in industrialized countries[10], and information on pedigrees going back many generations is difficult to collect reliably. For these reasons, investigators have recently begun looking at signatures of very distant inbreeding (e.g., common ancestry up to ~100 generations ago) using genome-wide single nucleotide polymorphism (SNP) data in an attempt to understand whether autozygosity increases the risk to schizophrenia and other complex diseases[11]. Autozygosity in SNP data is typically inferred from runs of homozygosity (ROHs): long, contiguous stretches (e.g., > 40) of homozygous SNPs. The proportion of the genome contained in such ROHs, *Froh*, can then be used to predict complex traits[12–19]. Keller et al.[11] showed that *Froh* is the optimal method for detecting inbreeding signals that are due to rare, recessive to partially recessive mutations, such as those thought to occur when traits are under directional selection[3]. The low variation in *Froh* means that large sample sizes (e.g., >12,000) are required to uncover realistic effects of distant inbreeding on complex diseases in samples unselected for inbreeding[11].

In 2012, Keller et al.[20] used the original Psychiatric Genomics Consortium schizophrenia data (17 case-control datasets, total *n* = 21,831) to investigate whether *Froh* is associated with increased risk of schizophrenia. The authors estimated that the odds of developing schizophrenia increased by approximately 17% for every additional percent of the genome that is contained in autozygous regions (β = 16.1, CI(β) = [6.93, 25.7], *p* = 6x10^-4^.) This was by far the largest study to that date examining the association between *Froh* and any psychiatric disorder, and the significant relationship between *Froh* and case-control status remained robust through secondary analyses of various covariate combinations, common vs. rare IBD haplotypes, and SNP thresholds used to define ROHs. These results are consistent with the hypothesis that autozygosity causally increases the risk of schizophrenia. Nevertheless, because various confounding factors may increase likelihood of distant inbreeding as well as the probability of having offspring with schizophrenia, these results do not imply a causal relationship. For example, parents higher on schizophrenia liability may pass their higher liability to offspring and mate with more genetically similar partners (e.g., due to decreased mobility, educational opportunities, etc.).

The current study seeks to provide a well-powered, independent replication of Keller et al.(2012)[20]. In light of the growing concern about publication bias[21,22] and dearth of well-powered replications[23,24], this follow-up analysis is a necessary step in validating the *Froh—*schizophrenia relationship. The present study used genome-wide SNP data from 22 independent schizophrenia case-control datasets (*n* = 39,830) from the PGC[25] to further examine the relationship between *Froh* and schizophrenia. Our replication attempt is an important contribution to the growing body of literature examining autozygosity and psychiatric disorders, and should help verify whether autozygosity estimated from ROHs is robustly related to schizophrenia risk and, by extension, can help elucidate whether schizophrenia risk alleles are biased, on average, toward recessive effects.

## Results

SNP data from 28,985 schizophrenia cases and 35,017 controls were collected as detailed in Ripke et al.[25]. Quality control (QC) and analyses were conducted separately for the original and replication datasets. The “original” dataset included subjects from the PGC’s SCZ1[26] samples used by Keller et al[20] (*n* = 21,868 after QC), and the “replication” dataset contained all subjects (*n* = 39,830 after QC) in the PGC SCZ2[25] samples not included in the original Keller et al. study, making the replication dataset independent of the original dataset analyzed in Keller et al.

Despite the number of imputed SNPs ranging from ~1.8 million to ~4.2 million in the datasets, there were not enough well imputed SNPs in common across all 22 datasets to conduct a viable ROH analysis in the same way as in the original study (see Methods). Nevertheless, Keller et al. also reported results from ROHs estimated from unimputed SNP data, and these results were highly consistent with imputed SNPs. Therefore, our primary analyses were conducted using post-QC, unimputed genotype data. We also report results on imputed SNPs (see S5–S12 Figs and S1 Table) using slightly different QC procedures than used in the original report (see Methods), which do not change the conclusions below. While ROHs from the imputed data were called from a common SNP set, ROHs from the unimputed data were called on unique sets of SNPs for each dataset.

Keller et al.[20] found that all ROH length thresholds were significantly associated with schizophrenia, but because ROH thresholds are ultimately arbitrary, they focused their discussion on the thresholds (e.g., 110 consecutive homozygous SNPs in the unimputed data) that maximized the schizophrenia-ROH relationship. In an attempt to follow as closely as possible the method used by Keller et al., we report two sets of ROH results. The first approach—a direct replication attempt of Keller et al.—defined ROHs as being ≥ 110 consecutive homozygous SNPs in a row (with median Mb ranging from ~1 to ~3.4 Mb, depending on sample) in the unimputed data. Because using unimputed SNP data introduces large differences in mean ROH length across datasets (when defined by number of consecutive homozygous SNPs) due to varying SNP densities, we also employed a secondary replication approach using a 2.3 Mb minimum length threshold that corresponds to 110 SNPs-in-a-row average length in the original report. As in the original report, we also show results across all thresholds to ensure that no results were missed.

Table 1 gives the descriptive statistics for average ROH lengths and *Froh* across datasets, where ROHs were defined as ≥ 110 consecutive homozygous SNPs. There was wide variation in average *Froh* and ROH lengths between datasets, a consequence of using unimputed SNP data, which introduces more between-dataset variability in *Froh* and mean ROH length[20]. Across datasets, mean *Froh* was also higher (0.30% vs. 0.14%) and average ROH lengths shorter (1.1–3.4 Mb vs. 2.0–4.7 Mb) in the replication versus original datasets. Part of the reason for the mean *Froh* discrepancy seemed to be due to replication datasets being genotyped on denser SNP chips, because this discrepancy reduced when we defined ROHs as ≥ 2.3 Mb homozygous SNPs (0.22% vs. 0.13%; Table 1). The remaining higher average *Froh* in the replication datasets appears to be due to more samples being from countries with higher overall *Froh* (e.g., Sweden, Estonia, Israel) in the replication datasets; the average *Froh* levels were very similar across replication vs. original datasets within the same countries.

**Table 1:** Descriptive data for the unimputed (post-QC) PGC replication data—ROHs defined as ≥ 110 consecutive homozygous SNPs or as ≥ 2.3 Mb long.

Dataset	N (post-QC)	N cases	Site	Platform	ROH definition: 110 SNPs-in-a-row				ROH definition: 2.3 Mb long			
					Avg Froh (*100)	SD Froh (*100)	Avg Mb	SD Mb	Avg Froh (*100)	SD Froh (*100)	Avg Mb	SD Mb
aarh	1699	841	Denmark	I650	0.22	0.70	2.35	3.12	0.16	0.66	4.42	4.34
ajsz	2484	891	Israel	I1M	0.85	0.92	2.36	2.62	0.56	0.89	4.50	3.52
asrb	664	395	Australia	I650	0.13	0.32	2.07	2.94	0.10	0.31	3.79	4.13
boco	2032	1214	Germany	Illum	0.14	0.50	2.61	3.72	0.11	0.50	4.38	4.82
clm2	5451	3358	UK	I1M	0.11	0.37	2.30	3.21	0.10	0.36	3.73	3.98
clo3	3638	2079	UK	omni	0.17	0.55	2.08	3.49	0.12	0.54	4.27	5.45
cou3	1186	508	UK	omni	0.13	0.24	1.80	3.06	0.08	0.24	3.41	4.92
egcu	1374	232	Estonia	omni	0.38	0.57	2.19	2.61	0.25	0.54	4.24	3.71
ersw	553	244	Sweden	omni	0.30	0.55	2.04	2.52	0.18	0.50	4.19	3.85
gras	2170	1041	Germany	AXI	0.25	0.73	2.00	2.58	0.15	0.67	4.73	3.92
irwt	2267	1277	Ireland	A6.0	0.17	0.23	2.14	1.93	0.14	0.22	3.59	2.22
lie2	399	130	US	O25	0.31	0.24	1.16	1.09	0.08	0.18	3.57	2.56
lie5	870	485	US	I550	0.13	0.24	1.98	1.55	0.09	0.20	3.52	1.75
msaf	436	308	US & Israel	A6.0	0.55	1.15	2.76	2.71	0.42	1.04	4.55	3.14
pewb	2327	566	Seven countries	I1M	0.13	0.44	2.27	2.52	0.11	0.40	3.88	3.09
pews	386	150	Spain	I1M	0.37	0.79	2.98	3.14	0.31	0.74	4.91	3.50
s234	3592	1558	Sweden	A6.0	0.28	0.53	2.38	2.44	0.21	0.46	4.03	3.03
swe5	4286	1723	Sweden	omni	0.30	0.64	2.32	3.27	0.21	0.61	4.46	4.75
swe6	2041	909	Sweden	omni	0.54	0.93	2.76	3.65	0.40	0.86	5.05	4.85
top8	206	139	Norway	A6.0	0.23	0.62	2.44	2.29	0.18	0.60	4.04	2.59
umeb	897	328	Sweden	omni	0.76	1.34	3.21	4.44	0.60	1.27	5.84	5.78
umes	872	186	Sweden	omni	1.03	1.26	3.43	4.03	0.84	1.22	5.64	4.81

### ROH burden results

**Fig 1:**
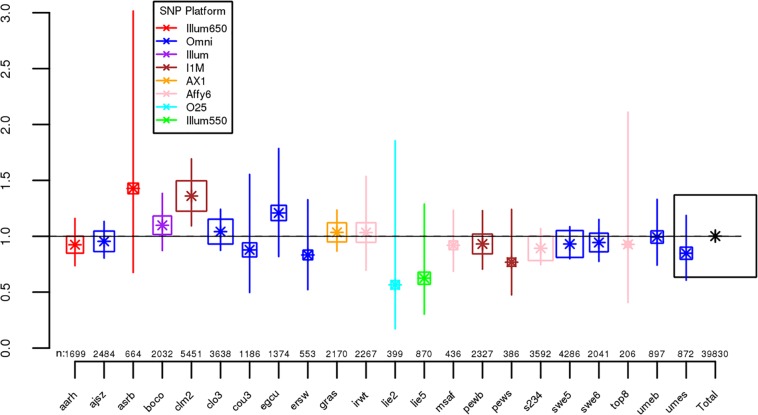
Estimated changes in odds of schizophrenia for each 1% increase in *Froh* (odds ratios; asterisks) and their 95% confidence intervals (bars) across the independent replication datasets (colored according to SNP platform) and for the total sample (black) from the unimputed SNP data, for ROHs defined as ≥ 110 consecutive homozygous SNPs. Boxes are proportional to the square root of sample sizes (also shown at the bottom). Dataset names are on the x-axis. Only one of the individual estimated odds ratios significantly differs from one (“clm2” dataset), and the overall effect (black) is *not* significant (β = 0.19, *Z* = 0.08, *p* = 0.94).

**Fig 2:**
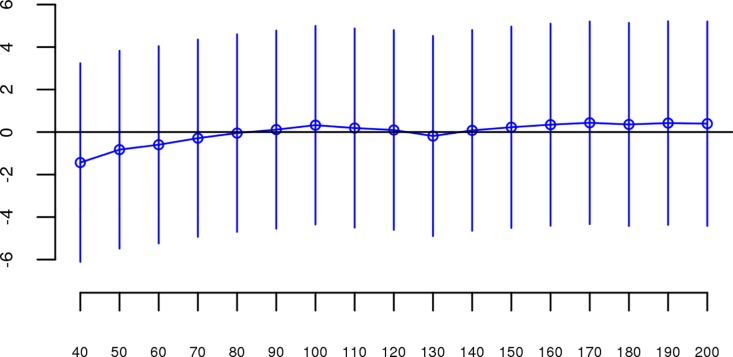
Slope estimates (the change in log odds for a 1% increase in *Froh*; points) and their 95% confidence intervals (bars) of *Froh* from unimputed SNP data predicting schizophrenia for different SNP thresholds of calling ROHs. No SNP homozygosity threshold was significant.

**Fig 3:**
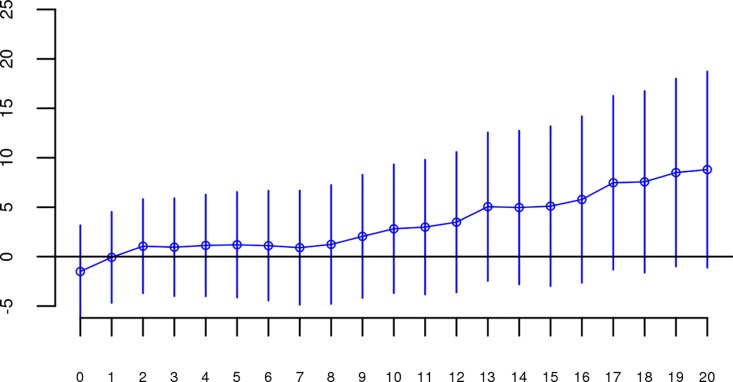
Slope estimates (the change in log odds for a 1% increase in *Froh*; points) and their 95% confidence intervals (bars) of *Froh* from unimputed SNP data predicting schizophrenia for different Mb thresholds of calling ROHs. No Mb length thresholds reached significance.

For each dataset, we regressed case-control status on *Froh* using mixed effects logistic regression treating dataset as a random factor, and controlled for 20 principal components (PCs) from the genomic relationship matrix[27] and two SNP quality measures (excess heterozygosity and SNP missingness; see Methods). In Keller et al. (2012), the authors used mixed effects models to test the ROH burden association with schizophrenia. However, in the current analysis we used fixed effect logistic regression models, treating dataset as a fixed, because a minority of the mixed effects models failed to converge. When the mixed effects models did converge, the results were highly similar to the respective fixed effect models. Figs 1 and S1 show the predicted change in odds of schizophrenia risk (and 95% confidence intervals) for every 1% increase in average *Froh* for each logistic regression in the replication data using ROHs defined by either ≥110 consecutive homozygous SNPs (Fig 1) or ROH length ≥ 2.3 Mb (S1 Fig). The overall association between schizophrenia and *Froh* in the replication data was in the predicted direction but not significant for ROHs defined as at least 110 consecutive homozygous SNPs (β = 0.19, CI(β) = [−4.50,4.88], *Z* = 0.08, *p* = 0.94) or for ROHs defined as ≥ 2.3 Mb (β = 0.75, CI(β) = [−4.05,5.56], *Z* = 0.31, *p* = 0.76). The results from analyses on ROHs called from imputed rather than raw SNP data were also non-significant (S5 Fig). As in Keller et al., we also explored increasingly long SNP and Mb ROH thresholds to assess the stability of the *Froh*-schizophrenia relationship (Figs 2 and 3). Across all thresholds, the only thresholds that approached significant associations between *Froh* and schizophrenia in the replication data were at the upper limits of the Mb-length ROH thresholds; the strongest association was for ROHs defined as ≥ 19 Mb (β = 8.64, CI(β) = [−0.85,18.13], *Z* = 1.78, *p* = 0.07).

We conducted a series of follow-up analyses to ensure that the failure to replicate our original report was not due to analytical error, inclusion of outlier individuals or datasets, or suppressing covariates in the replication data. We reran the same analyses described above on SNP data from the “original” report using the exact same quality control and analytic procedures performed on the replication data. Results were virtually identical to those obtained in Keller et al.’s 2012 study (S2–S4 Figs), increasing our confidence that the procedures used in the replication attempt were identical to those used in the original analysis and that the results from the original analysis were not due to analytic or procedural errors. We then reran analyses in the replication data after (a) omitting individuals with very long (>30 Mb) ROHs, (b) omitting only long ROHs, (c) including all combinations of covariates in the model (SNP missingness, average heterozygosity, 10 or 20 principle components), and (d) including only the longest ROH for each individual. The *Froh*-schizophrenia relationship remained non-significant in these follow-up analyses (results shown in S2 Table).

We noticed that there was greater variability in *Froh* in the replication datasets and that this greater variability was mostly driven by replication datasets that had *n* < 300. Under the premise that smaller samples might differ in genotypic or phenotypic quality, we excluded seven samples that contained fewer than 300 cases (“egcu”, “ersw”, “lie2”, “pews”, “top8”, “umes”), reran our baseline analysis (including all covariates mentioned above and using an ROH threshold of ≥ 110 consecutive homozygous SNPs), but still observed a non-significant *Froh-*schizophrenia relationship (β = 1.04, CI(β) = [−3.88,5.96], *Z* = 0.42, *p* = 0.68) in the predicted direction. Therefore, this post-hoc analysis does not lend support to the possibility that small samples in the replication set added noise to our analysis, obscuring an *Froh*-schizophrenia relationship.

**Fig 4:**
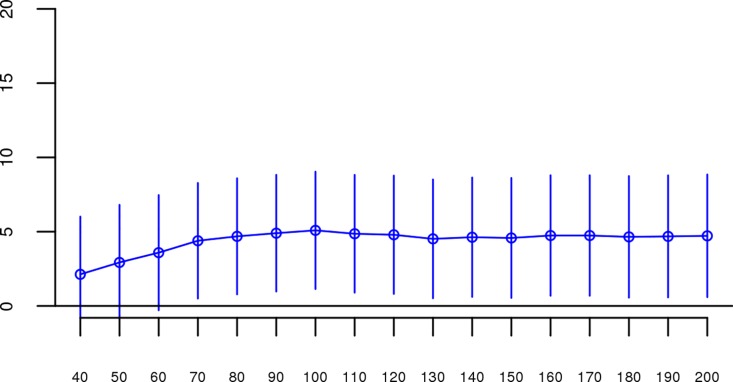
Slope estimates (the change in log odds for a 1% increase in *Froh*; points) and their 95% confidence intervals (bars) of *Froh* from the combined unimputed SNP data predicting schizophrenia for different SNP thresholds of calling ROHs. All SNP thresholds greater than 60 SNPs-in-a-row were significant.

**Fig 5:**
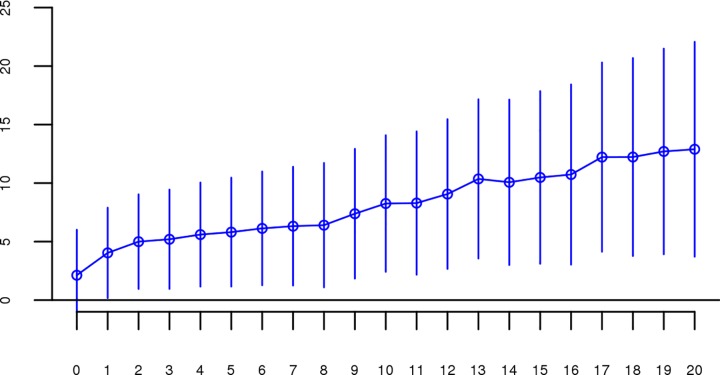
Slope estimates (the change in log odds for a 1% increase in *Froh*; points) and their 95% confidence intervals (bars) of *Froh* from the combined unimputed SNP data predicting schizophrenia for different Mb thresholds of calling ROHs. All length thresholds longer than 1 Mb were significant.

Although results from the replication analysis were not significant, they were in the same direction as the original analysis. It could therefore be argued that the best estimate of the association between ROHs and schizophrenia is obtained by combining the two datasets. When we reran our analyses on the combined original + replication data (n = 61,661), all *Froh* associations based on ROH thresholds greater than 60 consecutive homozygous SNPs or longer than 1 Mb were significant (Figs 4 and 5). For an ROH threshold of ≥ 110 consecutive homozygous SNPs), we observed a significant *Froh-*schizophrenia relationship in the combined data (β = 4.86, CI(β) = [0.90,8.83], *Z* = 2.40, *p* = 0.02). In this combined dataset, we also used a replication status-by-*Froh* interaction to conclude that the Froh-schizophrenia association was only marginally higher in the original compared to the replication datasets (interaction β = −3.98, *Z* = −1.84, *p* = 0.07) for ROHs defined as at least 110 consecutive homozygous SNPs.

### The effects of close versus distant inbreeding

**Fig 6:**
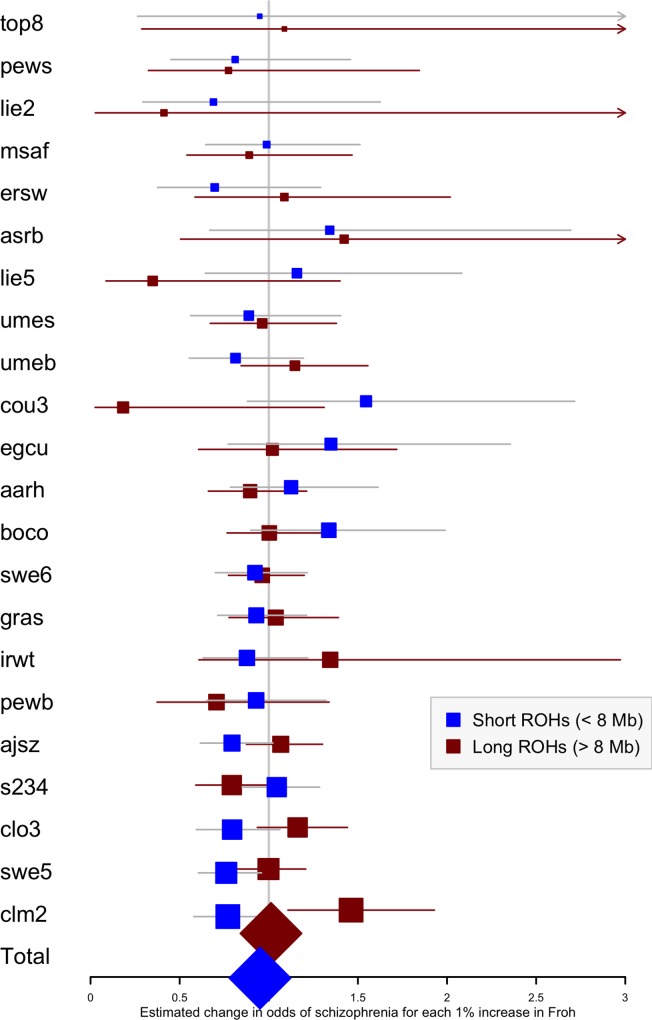
Forest plot of the change in odds of schizophrenia risk for each 1% increase in *Froh* due to short (< 8 Mb, blue) or long (> 8 Mb, red) ROHs for each sample in the replication. Boxes are proportional to the square root of sample sizes, and 95% confidence intervals are indicated by the horizontal lines. Dataset names are on the y-axis, with the estimates from the combined sample at the bottom.

To assess the relative importance of distant versus close inbreeding, we compared the effects of short versus long ROHs. As in the original study, we chose our ROH length threshold based on the Mb length cutoff that resulted in equal *Froh* variances, calculating *Froh_short* as the proportion of the genome contained in ROHs < 8 Mb long, and *Froh_long* as the proportion of the genome contained in ROHs > 8 Mb long. Although neither association was significant, the effect of *Froh*_*short* (β = −5.06, CI(β) = [−12.08,1.95], *Z* = −1.42, *p* = 0.16), caused by autozygosity arising from more ancient common ancestors, was negative (“protective”) and in the opposite direction of effect of *Froh*_*long* (β = 1.23, CI(β) = [−4.78,7.25], *Z* = 0.40, *p* = 0.69), caused by autozygosity arising from more recent common ancestors, which predicted increased risk for schizophrenia (Fig 6).

## Discussion

Despite exploring various homozygous SNP length thresholds, Mb thresholds, and combinations of covariates, the findings from this study do not lend much support to the original observation of a highly significant *Froh*-schizophrenia association[20], and provide only equivocal support, based on combining the original and replication data, for the hypothesis that autozygosity is a risk factor for schizophrenia.

Perhaps the simplest explanation for this pattern of results is that the conclusions about distant inbreeding from the original data represent a type-I error or that the lack of replication in the current report was a type-II error. Despite the fact that the effect in the original study was highly significant (*p* = 6x10^-4^) and the statistical power in the replication study to detect the observed effect size in the original study was nearly 100%, it is possible that the estimated effects of the original analysis could have been over-estimated and/or those of the replication analysis under-estimated, due to sampling variability. There is some support for this interpretation, as there was not a significant difference in results between replication versus original datasets (interaction *p* = 0.07).

An alternative explanation for the overall pattern of results has to do with the potential influence of unmeasured confounding factors in both the original and replication analyses. Unlike genotype frequencies, which change very slowly and are unaffected by inbreeding, ROH levels can change substantially after even a single generation of inbreeding, making ROH analyses highly susceptible to confounding factors associated with both disease risk and the degree of inbreeding/outbreeding. For example, contrary to initial predictions, Abdellaoui et al.[28] identified a significant and negative (“protective”) relationship between *Froh* and risk for major depressive disorder (MDD) in the Dutch population. However, the authors found that religiosity was significantly associated with both higher autozygosity and lower MDD in this population. When religiosity was accounted for in their regression model, the original association between MDD and *Froh* disappeared. A similar effect was detected for educational attainment: highly educated individuals were more likely to migrate and mate with highly educated and more diverse partners, making highly educated spouse pairs share less ancestry and leading to their offspring having lower *Froh*[29]. Thus, assortative mating on variables such as education or religion could subtly influence observed *Froh* associations, potentially affecting results in ways that can be difficult to account for. For example, an observed *Froh*-schizophrenia relationship could be due to parents with a higher schizophrenia liability mating with less genetically diverse mates due to, e.g., fewer educational opportunities or lower migration rates. Thus, the causation may be reversed: schizophrenia liability in parents could cause not only higher schizophrenia risk, but also higher *Froh*, in offspring rather than *Froh* in offspring increasing their schizophrenia liability. Such reverse and third variable causation possibilities can only be tested if relevant socio-demographic variables in subjects and (optimally) their parents are collected.

The possibility of unmeasured variables confounding *Froh-*disorder relationships seems particularly likely in analyses conducted on ascertained samples. Ascertainment of cases and controls not perfectly matched on socio-demographic factors that might affect degree of outbreeding (e.g., socioeconomic status, education level, age, religion, urbanicity) can mask any true *Froh* association and bias the observed association in either direction. Such a scenario might explain otherwise contradictory findings in previous ROH case-control analyses[18,28,30–36]. For example, following two studies showing that genome-wide autozygosity was significantly associated with schizophrenia risk, including the original Keller et al. study[13,20], two newer studies failed to replicate this association[34,35], although both replication sample sizes (*n* = 3,400 and 11,244 respectively) were substantially smaller than the current one (*n =* 39,830). (It should be noted that the sample used in the latter study[36] overlapped with the samples in both the original Keller et al.[20] study and the current replication study). Even within the same study, *Froh* results in ascertained samples have been inconsistent. Using PGC MDD data, Power et al.[36] found a significant positive *Froh*-MDD relationship in data from three German sites but a significant *negative Froh*-MDD relationship in six non-German sites. A possible explanation for this and other such examples of heterogeneity across sites they observed is that cases and controls differed on socio-demographic factors that were associated with *Froh*, and the direction of this ascertainment bias was inconsistent across data collection sites.

We believe that similar ascertainment biases could have affected results in the present study as well as in the original Keller et al.[20] report. Many of the PGC schizophrenia datasets used cases ascertained from hospitals, clinics, health surveys, and advertisements but controls from previous biomedical research volunteers, university students, blood donors, and population registries. While such differences in ascertainment between cases and controls are highly unlikely to lead to allele frequency differences, and thus are of little concern to genome-wide association studies, they could very easily lead to *Froh* differences due to differences in degree of inbreeding/outbreeding in the populations from which cases and controls were drawn. Controlling for ancestry principal components in this case would only help to the degree that degree of inbreeding/outbreeding is associated with ancestry. Unfortunately, none of the other variables that might statistically control for such biases due to differences in case/control ascertainment are currently available in the PGC data collection. The PGC collection of studies was designed for association analyses; it was not optimally designed for ancillary purposes, such as ROH analyses.

It is important to recognize that even ascertainment biases that differ at random across sites would substantially inflate type-I error rates because the proper degrees of freedom for the test should be closer to the number of independent sites rather than the number of independent cases and controls. To demonstrate this, we permuted data under the null hypothesis of no relationship between *Froh* and schizophrenia in the 17 datasets from the original 2012 study by randomly flipping case or control status within each dataset for each permutation (e.g., cases and control statuses in a dataset either remained the same or were flipped to the opposite status). We then calculated the overall *Froh ~* schizophrenia relationship with the same logistic regression model and using the same covariates as in the original analysis. Across 1,000 permutations, 183 p-values were significant (*p* < 0.05), implying a type-I error rate of 0.18 and demonstrating how false conclusions about *Froh* relationships can be reached even when ascertainment biases are random across multiple sites.

## Conclusion

Given concerns about the false discovery rate in science[22], there has been increasing emphasis on the need for well-powered, direct replications of novel findings in genetics[23,37,38] and other fields[39–41]. The current study was a well-powered, direct replication attempt that failed to replicate an earlier finding that autozygosity arising from distant common ancestors was significantly associated with schizophrenia. As is typical with null findings, it is difficult to identify the reason for this failure to replicate. However, we have argued that a likely cause is that ROH associations are highly susceptible to confounding, especially in case-control (ascertained) samples. Thus, we believe that the conclusions of the original study were premature and the true causal relationship between schizophrenia and autozygosity could be either stronger/more positive (if the populations from which controls were ascertained were, on average, slightly less outbred than populations from which cases were ascertained) or weaker/more negative (the reverse) than reported here. Unfortunately, we do not have the ability to test these hypotheses directly in the current datasets, and doing so awaits either new samples in which cases and controls are carefully matched or the collection of information that allows potential confounders to be statistically controlled. This creates a dilemma for ROH analyses using existing case-control genome-wide data: GWAS datasets usually do not match cases and controls to the degree necessary to rule out confounding effects on ROH analyses and typically do not collect the relevant socio-demographic information necessary to control for potential confounders. The current study therefore serves as a cautionary tale for analyzing ROHs in existing ascertained GWAS datasets. Such datasets may be perfectly adequate for their designed purpose–GWAS–but may be problematic and even misleading for ROH analyses.

## Methods

### Psychiatric Genomics Consortium GWAS Data

Our study used 37 datasets from the Psychiatric Genomics Consortium’s SCZ2 data–these data included 28,985 schizophrenia cases and 35,017 controls, collected from 37 sites in 13 countries. Data collection and ascertainment details are described elsewhere.[25]

Keller et al.[20] used 17 datasets from the PGC SCZ1[26] data. Several of these original 17 studies recruited additional subjects by the time of our study, necessitating two well-defined, independent datasets: one including all of the individuals analyzed in the original 2012 study (“original” dataset), and one containing only subjects not included in Keller et al.’s 2012 report (the “replication” dataset, comprised of 22 studies and a total sample size of 18,562 cases and 21,268 controls after QC; see Table 1). Three of the original case-control datasets from the PGC’s SCZ1 added more subjects and/or controls in SCZ2, but only two of these datasets had enough subjects to pass QC and merit inclusion in the current study—thus there is a “top8” dataset (N = 180) in this replication study, comprised of the samples that were added to the “top3” dataset (N = 598) from the original 2012 study, and a “boco” dataset (N = 1,870), which includes the new cases and controls that were added to the original “bon” dataset (N = 1,778). For consistency with the original Keller et al. (2012) study[20], we excluded the three family-based datasets of parent-proband trios and three East Asian datasets.

### Quality Control (QC) Procedures–Raw SNP Data

We followed the same QC procedures as Keller et al.[20]. We removed a) one individual from any pair of individuals who were related with $\hat{\pi }$ >0.2, b) individuals with non-European ancestry as determined by principal components analysis; c) samples with SNP missingness >0.02; or d) samples with genome-wide heterozygosities >6 standard deviations above the mean. SNPs were excluded if they a) deviated from Hardy-Weinberg equilibrium at p<1×10^−6^; b) had missingness >0.02; or c) had a missingness difference between cases and controls >0.02.

### QC Procedures–Imputed SNP Data

Early in the analysis process, we found that only including SNPs with imputation dosage r^2^ > .90 across all datasets, as was done in the original study[20], left us with too few SNPs with which to conduct viable ROH analyses in the replication data. Because having ROHs of similar length and SNP density is important for comparing present results to those from the 2012 study, we decided that having a similar number of SNPs to Keller et al.[20] was more important than following the exact same QC procedures. Thus, to arrive at a similar number of genome-wide SNPs in the new and old datasets, some of the QC measures described below were different than in the 2012 investigation.

SNPs were imputed using the 1000 Genomes reference panel[42]; imputation procedures are described elsewhere[25]. Imputation dosages were converted to best-guess (highest posterior probability) SNP calls because ROH detection algorithms require discrete SNP calls, and extremely stringent QC thresholds were employed to achieve accuracy rates similar to those in genotyped SNPs[43]. We excluded any imputed SNPs that were not included in the HapMap3[44] reference panel, as done in the 2012 study. Unlike the original QC procedures, we did not require that the dosage r^2^ had to be > .90 in each individual datasets. We excluded any imputed SNPs that had a dosage r^2^<0.98 or >1.02 in the overall sample (calculated using average dosage r^2^ weighted by sample size) or that had MAF<0.15 within each sample (vs. .05 in original), leaving 340,084 high-quality imputed SNPs (vs. 398,325 in original).

### ROH Calling Procedures

Again, we followed the same ROH calling procedures as in Keller et al[20]. As recommended in a separate investigation[45] by three of the authors of the present study, we chose PLINK software[46] for its computational efficiency and superior detection of autozygous stretches. As in the 2012 study, we pruned for LD using PLINK’s—indep flag, which ensures more uniform SNP coverage across the genome and reduces false autozygosity calls by removing redundant markers. We pruned SNPs for LD using a VIF threshold of 10, which is equivalent to multiple R^2^ > 0.90 between the focal SNP and the 50 surrounding SNPs.

We called ROHs using PLINK’s—homozyg flags, defining initial ROHs as being ≥40 homozygous SNPs in a row with no heterozygote calls allowed. We required that ROHs have a density greater than 1 SNP per 200 kb, and split an ROH into two if a gap >500 kb existed between consecutive homozygous SNPs. We then post-processed the initial ROH calls by altering the SNPs-in-a-row threshold and the Mb length threshold; specifically, we looked at ROH calls with a minimum of 40 to 200 consecutive homozygous SNPs in increments of 10, and ROH calls with minimum lengths ranging from 1 to 20 Mb by increments of 1 Mb. We varied ROH thresholds this widely to ensure that no potential effects of autozygosity were missed, but the primary results presented here are based on two replication attempts in the unimputed data: (a) using the same SNP thresholds that gave the most straightforward comparison with the original report (this was 110 SNPs-in-a-row for the unimputed data, spanning ~1 to ~2.1 Mb in the replication datasets, and 65 SNPs-in-a-row for the imputed data), and (b) using the physical length threshold (2.3 Mb) that corresponded to the average Mb length for 110 SNPs-in-a row in the original report.

### ROH Burden Analysis

After calling ROHs, we summed the total length of all autosomal ROHs for each individual and divided that by the total SNP-mappable distance (2.77x10^9^ bases) to calculate *Froh*. *Froh*, the proportion of the genome contained in long homozygous regions, was used as the predictor of schizophrenia case-control status in analyses described below. As confounding factors such as population stratification, SNP missingness, call quality, and plate effects can influence *Froh*, we included the first 20 principle components (based on a genome relationship matrix calculated from ~30K LD-pruned SNPs), percentage of missing SNP calls in the raw data, and excess heterozygosity in all regression models[20]. We then regressed case-control status on *Froh* using a mixed linear effects logistic regression model (available in the lme4 package in R version 3.1.0), treating dataset as a random factor, to assess the overall effect of *Froh* on schizophrenia across all sites. Some of the models with random effects did not converge; thus, for consistency, we modeled dataset as a fixed factor for all analyses. The results from mixed linear effects models that converged were very similar to fixed effects models, giving us confidence that the fixed effects results of this analysis and the random effect results from the original Keller et al. (2012) study are commensurate. We also ran logistic regressions in each of the 22 datasets separately.

### Ethics Statement

This research was approved by CU Boulder's Institutional Review Board with regard to protocol number 13–0266 on 3/29/2016 in accordance with Federal Regulations at 45 CFR 46. Written patient consent was obtained for each individual study by the study PI, with the exception of the "clm3" and "clo3" datasets, which obtained anonymous samples via a drug monitoring service under ethical approval and in accordance with the UK Human Tissue Act.

## Supporting Information

S1 Table Descriptives for the imputed independent PGC replication data, for ROHs defined as 65 SNPs or greater. (DOCX)

S2 Table Results from follow-up analyses to ensure that failure to replicate was not due to inclusion of outlier individuals or datasets, or suppressing covariates in the replication data. (DOCX)

S1 Fig Estimated changes in odds of schizophrenia for each 1% increase in *Froh* (odds ratios; asterisks) and their 95% confidence intervals (bars) across the independent replication data (colored) and for the total sample (black) from the unimputed SNP data, for ROHs defined as ≥ 2.3 Mb. Boxes are proportional to the square root of sample sizes (also shown at the bottom). Dataset names are on the x-axis. Only one of the individual estimated odds ratios significantly differs from one (“clm2” dataset), and the overall effect (black) is not significant (β = 0.75, *Z* = 0.31, *p* = 0.76).
(TIFF)

S2 Fig Estimated changes in odds of schizophrenia for each 1% increase in *Froh* (odds ratios; asterisks) and their 95% confidence intervals (bars) across the original PGC SCZ1 data (colored) and for the total sample (black) from the unimputed SNP data. Boxes are proportional to the square root of sample sizes (also shown at the bottom). Dataset names are on the x-axis. (While the y-axis is cut off at 3 for clarity, it should be noted that the upper limit of the 95% confidence interval is 4.1 for the “muc” dataset and 5.4 for the “top3” dataset.) Only one of the individual estimated odds ratios significantly differ from one (the “muc” dataset), but the overall effect (black) is significant (Beta = 16.83, *p* = 0.000357.)
(TIFF)

S3 Fig Slope estimates (the change in log odds for a 1% increase in *Froh*; points) and their 95% confidence intervals (bars) of *Froh* from unimputed PGC SCZ1 original SNP data predicting schizophrenia for different SNP thresholds of calling ROHs. All SNP homozygosity thresholds above 40 SNPs-in-a-row were significant.
(TIFF)

S4 Fig Slope estimates (the change in log odds for a 1% increase in *Froh*; points) and their 95% confidence intervals (bars) of *Froh* from unimputed PGC SCZ1 original SNP data predicting schizophrenia for different Mb thresholds of calling ROHs. All ROH Mb thresholds equal to and longer than 1 Mb were significant.
(TIFF)

S5 Fig Estimated changes in odds of schizophrenia for each 1% increase in *Froh* (odds ratios; asterisks) and their 95% confidence intervals (bars) across the independent replication datasets (colored) and for the total sample (black) from the imputed SNP data, for ROHs defined as ≥ 65 homozygous SNPs in a row. Boxes are proportional to the square root of sample sizes (also shown at the bottom). Dataset names are on the x-axis. Only one of the individual estimated odds ratios significantly differs from one (“clm2” dataset), and the overall effect (black) is *not* significant (β = 0.11, *Z* = 0.05, *p* = 0.96).
(TIFF)

S6 Fig Slope estimates (the change in log odds for a 1% increase in *Froh*; points) and their 95% confidence intervals (bars) of *Froh* from imputed PGC SCZ2 replication SNP data predicting schizophrenia for different SNP thresholds of calling ROHs. No SNP homozygosity thresholds were significant.
(TIFF)

S7 Fig Slope estimates (the change in log odds for a 1% increase in *Froh*; points) and their 95% confidence intervals (bars) of *Froh* from imputed PGC SCZ2 replication SNP data predicting schizophrenia for different Mb thresholds of calling ROHs. No ROH Mb thresholds were significant.
(TIFF)

S8 Fig Estimated changes in odds of schizophrenia for each 1% increase in *Froh* (odds ratios; asterisks) and their 95% confidence intervals (bars) across the original PGC SCZ1 datasets (colored) and for the total sample (black) from the imputed SNP data for SNPs defined as ≥ 65 homozygous SNPs in a row. Boxes are proportional to the square root of sample sizes (also shown at the bottom). Dataset names are on the x-axis—note that this imputed analysis was performed on the original SCZ1 *individuals* but within the PGC’s SCZ2 data, where some of the original individuals were divided among several new datasets. This is why some of the dataset names are slightly different from those in the original unimputed PGC SCZ1 data in S2 Fig. Only one of the individual estimated odds ratios significantly differs from one, the “munc” dataset, but the overall effect (black) is significant (Beta = 14.88, *Z* = 2.43, *p* = 0.02.)
(TIFF)

S9 Fig Slope estimates (the change in log odds for a 1% increase in *Froh*; points) and their 95% confidence intervals (bars) of *Froh* from imputed PGC SCZ1 original SNP data predicting schizophrenia for different SNP thresholds of calling ROHs. All SNP length thresholds were significant.
(TIFF)

S10 Fig Slope estimates (the change in log odds for a 1% increase in *Froh*; points) and their 95% confidence intervals (bars) of *Froh* from imputed PGC SCZ1 original SNP data predicting schizophrenia for different Mb thresholds of calling ROHs. All ROH Mb thresholds were significant.
(TIFF)

S11 Fig Slope estimates (the change in log odds for a 1% increase in *Froh*; points) and their 95% confidence intervals (bars) of *Froh* from the combined imputed SNP data predicting schizophrenia for different SNP thresholds of calling ROHs. SNP thresholds of 120 homozygous SNPs-in-a-row and above were significant.
(TIFF)

S12 Fig Slope estimates (the change in log odds for a 1% increase in *Froh*; points) and their 95% confidence intervals (bars) of *Froh* from the combined imputed SNP data predicting schizophrenia for different Mb thresholds of calling ROHs. All Mb thresholds ≥ 3 Mb were significant
(TIFF)

## References

[1] Darwin C. The effects of cross and self fertilisation in the vegetable kingdom. J. Murray; 1876.

[2] Walsh B. Evolutionary Quantitative Genetics. Handbook of Statistical Genetics: Third Edition. 2008 p. 533–86.

[3] Charlesworth B, Charlesworth D. The genetic basis of inbreeding depression. Genet Res. 1999;74(3):329–40. 10689809 10.1017/s0016672399004152

[4] Abaskuliev AA, Skoblo G V. Inbreeding, endogamy and exogamy among relatives of schizophrenia patients. Genetika. 1975;11(3):145–8. 135710

[5] Bulaeva OA, Pavlova TA, Bulaeva KB. The effect of inbreeding on accumulation of complex diseases in genetic isolates. Genetika. 2009;45(8):1096–104. 19769299

[6] Mansour H, Fathi W, Klei L, Wood J, Chowdari K, Watson A, et al Consanguinity and increased risk for schizophrenia in Egypt. Schizophr Res. 2010;120(1–3):108–12. 10.1016/j.schres.2010.03.026 20435442 PMC2900407

[7] Chaleby K, Tuma TA. Cousin marriages and schizophrenia in Saudi Arabia. Br J Psychiatry. 1987;150(4.):547–9. 3664138 10.1192/bjp.150.4.547

[8] Gindilis VM, Gaĭnullin RG, Shmaonova LM. Genetico-demographic patterns of the prevalence of various forms of endogenous psychoses. Genetika. 1989;25(4):734–43. 2759447

[9] Rudan I, Rudan D, Campbell H, Carothers A, Wright A, Smolej-Narancic N, et al Inbreeding and risk of late onset complex disease. J Med Genet. 2003 12 1;40 (12): 925–32. 10.1136/jmg.40.12.925 14684692 PMC1735350

[10] Bittles AH, Neel J V. The costs of human inbreeding and their implications for variations at the DNA level. Nat Genet. 1994;8(2):117–21. 10.1038/ng1094-117 7842008

[11] Keller MC, Visscher PM, Goddard ME. Quantification of inbreeding due to distant ancestors and its detection using dense single nucleotide polymorphism data. Genetics. 2011;189(1):237–49. 10.1534/genetics.111.130922 21705750 PMC3176119

[12] Vine AE, McQuillin A, Bass NJ, Pereira A, Kandaswamy R, Robinson M, et al No evidence for excess runs of homozygosity in bipolar disorder. Psychiatr Genet. 2009;19(4):165–70. 10.1097/YPG.0b013e32832a4faa 19451863

[13] Lencz T, Lambert C, DeRosse P, Burdick KE, Morgan TV, Kane JM, et al Runs of homozygosity reveal highly penetrant recessive loci in schizophrenia. Proc Natl Acad Sci U S A. 2007;104(50):19942–7. 10.1073/pnas.0710021104 18077426 PMC2148402

[14] Ku CS, Naidoo N, Teo SM, Pawitan Y. Regions of homozygosity and their impact on complex diseases and traits. Human Genetics. 2011 p. 1–15. 10.1007/s00439-010-0920-6 21104274

[15] McQuillan R, Leutenegger AL, Abdel-Rahman R, Franklin CS, Pericic M, Barac-Lauc L, et al Runs of Homozygosity in European Populations. Am J Hum Genet. 2008;83(3):359–72. 10.1016/j.ajhg.2008.08.007 18760389 PMC2556426

[16] Kirin M, McQuillan R, Franklin CS, Campbell H, Mckeigue PM, Wilson JF. Genomic runs of homozygosity record population history and consanguinity. PLoS One. 2010;5(11). 10.1371/journal.pone.0013996 PMC2981575 21085596

[17] Enciso-Mora V, Hosking FJ, Houlston RS. Risk of breast and prostate cancer is not associated with increased homozygosity in outbred populations. Eur J Hum Genet. 2010;18(8):909–14. 10.1038/ejhg.2010.53 20407466 PMC2987391

[18] Spain SL, Cazier J-B, Houlston R, Carvajal-Carmona L, Tomlinson I. Colorectal cancer risk is not associated with increased levels of homozygosity in a population from the United Kingdom. Cancer Res. 2009;69(18):7422–9. 10.1158/0008-5472.CAN-09-0659 19723657

[19] Hosking FJ, Papaemmanuil E, Sheridan E, Kinsey SE, Lightfoot T, Roman E, et al Genome-wide homozygosity signatures and childhood acute lymphoblastic leukemia risk. Blood. 2010;115(22):4472–7. 10.1182/blood-2009-09-244483 20231427

[20] Keller MC, Simonson MA, Ripke S, Neale BM, Gejman P V., Howrigan DP, et al Runs of homozygosity implicate autozygosity as a schizophrenia risk factor. PLoS Genet. 2012;8(4). 10.1371/journal.pgen.1002656 PMC3325203 22511889

[21] Thornton A, Lee P. Publication bias in meta-analysis: Its causes and consequences. J Clin Epidemiol. 2000;53(2):207–16. 10729693 10.1016/s0895-4356(99)00161-4

[22] Ioannidis JPA., Ioannidis JPA. Why most published research findings are false. PLoS Med. 2005;2(8):e124 10.1371/journal.pmed.0020124 16060722 PMC1182327

[23] Duncan LE, Keller MC. A critical review of the first 10 years of candidate gene-by-environment interaction research in psychiatry. American Journal of Psychiatry. 2011 p. 1041–9. 10.1176/appi.ajp.2011.11020191 21890791 PMC3222234

[24] Collaboration OS. Estimating the reproducibility of psychological science. Sci. 2015 8 28;349 (6251). 10.1126/science.aac4716 26315443

[25] Ripke S, Neale BM, Corvin A, Walters JTR, Farh K-H, Holmans P a., et al Biological insights from 108 schizophrenia-associated genetic loci. Nature. 2014;511:421–7. 10.1038/nature13595 25056061 PMC4112379

[26] Ripke S, Sanders AR, Kendler KS, Levinson DF, Sklar P, Holmans PA, et al Genome-wide association study identifies five new schizophrenia loci. Nat Genet. 2011;43(10):969–76. 10.1038/ng.940 21926974 PMC3303194

[27] Price AL, Patterson NJ, Plenge RM, Weinblatt ME, Shadick NA, Reich D. Principal components analysis corrects for stratification in genome-wide association studies. Nat Genet. 2006;38(8):904–9. 10.1038/ng1847 16862161

[28] Abdellaoui A, Hottenga JJ, Xiao X, Scheet P, Ehli EA, Davies GE, et al Association between autozygosity and major depression: Stratification due to religious assortment. Behav Genet. 2013;43(6):455–67. 10.1007/s10519-013-9610-1 23978897 PMC3827717

[29] Abdellaoui A, Hottenga JJ, Willemsen G, Bartels M, Van Beijsterveldt T, Ehli EA, et al Educational attainment influences levels of homozygosity through migration and assortative mating. PLoS One. 2015;10(3). 10.1371/journal.pone.0118935 PMC4347978 25734509

[30] Nalls MA, Guerreiro RJ, Simon-Sanchez J, Bras JT, Traynor BJ, Gibbs JR, et al Extended tracts of homozygosity identify novel candidate genes associated with late-onset Alzheimer’s disease. Neurogenetics. 2009;10(3):183–90. 10.1007/s10048-009-0182-4 19271249 PMC2908484

[31] Sims R, Dwyer S, Harold D, Gerrish A, Hollingworth P, Chapman J, et al No evidence that extended tracts of homozygosity are associated with Alzheimer’s disease. Am J Med Genet Part B Neuropsychiatr Genet. 2011;156(7):764–71. 10.1002/ajmg.b.31216 21812096

[32] Ghani M, Sato C, Lee JH, Reitz C, Moreno D, Mayeux R, et al Evidence of recessive Alzheimer disease loci in a Caribbean Hispanic data set: genome-wide survey of runs of homozygosity. JAMA Neurol. 2013;70(10):1261–7. 10.1001/jamaneurol.2013.3545 23978990 PMC3991012

[33] Assié G, LaFramboise T, Platzer P, Eng C. Frequency of germline genomic homozygosity associated with cancer cases. Jama. 2008;299(12):1437–45. 10.1001/jama.299.12.1437 18364486

[34] Ruderfer DM, Lim ET, Genovese G, Moran JL, Hultman CM, Sullivan PF, et al No evidence for rare recessive and compound heterozygous disruptive variants in schizophrenia. Eur J Hum Genet. 2014;23(July):1–3. 25370044 10.1038/ejhg.2014.228 PMC4666583

[35] Heron EA, Cormican P, Donohoe G, O’Neill FA, Kendler KS, Riley BP, et al No evidence that runs of homozygosity are associated with schizophrenia in an Irish genome-wide association dataset. Schizophr Res. 2014;154(1–3):79–82. 10.1016/j.schres.2014.01.038 24560374 PMC4034753

[36] Power RA, Keller MC, Ripke S, Abdellaoui A, Wray NR, Sullivan PF, et al A recessive genetic model and runs of homozygosity in major depressive disorder. Am J Med Genet Part B Neuropsychiatr Genet. 2014;165(2):157–66. 10.1002/ajmg.b.32217 PMC4234115 24482242

[37] Sullivan PF. Spurious Genetic Associations. Biol Psychiatry. 2007;61(10):1121–6. 10.1016/j.biopsych.2006.11.010 17346679

[38] Collins AL, Kim Y, Sklar P, O’Donovan MC, Sullivan PF. Hypothesis-driven candidate genes for schizophrenia compared to genome-wide association results. Psychol Med. Cambridge Univ Press; 2012;42(03):607–16. 21854684 10.1017/S0033291711001607 PMC4188923

[39] Button KS, Ioannidis JP a, Mokrysz C, Nosek B a, Flint J, Robinson ESJ, et al Power failure: why small sample size undermines the reliability of neuroscience. Nat Rev Neurosci. 2013;14(5):365–76. 10.1038/nrn3475 23571845

[40] Peng RD. Reproducible research and Biostatistics. Biostatistics. Biometrika Trust; 2009;10(3):405–8. 10.1093/biostatistics/kxp014 19535325

[41] Makel MC, Plucker JA, Hegarty B. Replications in psychology research how often do they really occur? Perspect Psychol Sci. Sage Publications; 2012;7(6):537–42. 10.1177/1745691612460688 26168110

[42] The 1000 Genomes Project Consortium. An integrated map of genetic variation from 1,092 human genomes. Nature. 2012 11 1;491(7422):56–65. 10.1038/nature11632 23128226 PMC3498066

[43] Hao K, Chudin E, McElwee J, Schadt EE. Accuracy of genome-wide imputation of untyped markers and impacts on statistical power for association studies. BMC Genet. 2009;10:27 10.1186/1471-2156-10-27 19531258 PMC2709633

[44] Consortium IH. A haplotype map of the human genome. Nature. Nature Publishing Group; 2005;437(7063):1299–320. 10.1038/nature04226 16255080 PMC1880871

[45] Howrigan DP, Simonson MA, Keller MC. Detecting autozygosity through runs of homozygosity: A comparison of three autozygosity detection algorithms. BMC Genomics. 2011 p. 460 10.1186/1471-2164-12-460 21943305 PMC3188534

[46] Purcell S, Neale B, Todd-Brown K, Thomas L, Ferreira MAR, Bender D, et al PLINK: a tool set for whole-genome association and population-based linkage analyses. Am J Hum Genet. 2007;81(3):559–75. 10.1086/519795 17701901 PMC1950838

