# Supplementary material for: No Reliable Association between Runs of Homozygosity and Schizophrenia in a Well-Powered Replication Study
Source: PLoS Genet. 2016 Oct 28;12(10):e1006343. doi: 10.1371/journal.pgen.1006343 (PMC5085024; doi:10.1371/journal.pgen.1006343)
Supplement: S1 Table — (DOCX) [file pgen.1006343.s001.docx]

**Table S1. Descriptives for the imputed independent PGC replication data, for ROHs defined as 65 SNPs or greater.**

| **Dataset** | **N** | **N cases** | **Site** | **Platform** | **Avg Froh(*100)** | **SD Froh(*100)** | **Avg Mb** | **SD Mb** |
| --- | --- | --- | --- | --- | --- | --- | --- | --- |
| aarh | 1699 | 841 | Denmark | I650 | 0.19 | 0.66 | 2.86 | 3.49 |
| ajsz | 2484 | 891 | Israel | I1M | 0.67 | 0.86 | 3.38 | 3.14 |
| asrb | 664 | 395 | Australia | I650 | 0.12 | 0.30 | 2.48 | 3.90 |
| boco | 2032 | 1214 | Germany | Illum | 0.14 | 0.49 | 2.83 | 3.71 |
| clm2 | 5451 | 3358 | UK | I1M | 0.12 | 0.35 | 2.45 | 2.91 |
| clo3 | 3638 | 2079 | UK | omni | 0.14 | 0.52 | 2.68 | 3.92 |
| cou3 | 1186 | 508 | UK | omni | 0.11 | 0.23 | 2.22 | 3.34 |
| egcu | 1374 | 232 | Estonia | omni | 0.32 | 0.54 | 2.82 | 3.06 |
| ersw | 553 | 244 | Sweden | omni | 0.24 | 0.50 | 2.77 | 3.14 |
| gras | 2170 | 1041 | Germany | AXI | 0.21 | 0.71 | 3.39 | 4.70 |
| irwt | 2267 | 1277 | Ireland | A6.0 | 0.17 | 0.23 | 2.52 | 2.36 |
| lie2 | 399 | 130 | US | O25 | 0.11 | 0.19 | 2.39 | 2.19 |
| lie5 | 870 | 485 | US | I550 | 0.13 | 0.24 | 2.52 | 2.27 |
| msaf | 436 | 308 | US & Israel | A6.0 | 0.50 | 1.14 | 3.99 | 4.07 |
| pewb | 2327 | 566 | Seven countries | I1M | 0.14 | 0.43 | 2.58 | 2.67 |
| pews | 386 | 150 | Spain | I1M | 0.36 | 0.77 | 3.91 | 4.18 |
| s234 | 3592 | 1558 | Sweden | A6.0 | 0.27 | 0.52 | 2.87 | 2.96 |
| swe5 | 4286 | 1723 | Sweden | omni | 0.26 | 0.61 | 2.96 | 3.82 |
| swe6 | 2041 | 909 | Sweden | omni | 0.46 | 0.86 | 3.48 | 3.89 |
| top8 | 206 | 139 | Norway | A6.0 | 0.23 | 0.61 | 2.86 | 3.17 |
| umeb | 897 | 328 | Sweden | omni | 0.67 | 1.27 | 4.17 | 5.39 |
| umes | 872 | 186 | Sweden | omni | 0.93 | 1.21 | 4.23 | 4.62 |
