# Supplementary material for: No Reliable Association between Runs of Homozygosity and Schizophrenia in a Well-Powered Replication Study
Source: PLoS Genet. 2016 Oct 28;12(10):e1006343. doi: 10.1371/journal.pgen.1006343 (PMC5085024; doi:10.1371/journal.pgen.1006343)
Supplement: S2 Table — (DOCX) [file pgen.1006343.s002.docx]

**Table S2. Results from follow-up analyses to ensure that failure to replicate was not due to inclusion of outlier individuals or datasets, or suppressing covariates in the replication data.**

| **Follow-up Analysis** | ***Froh* Beta** | **95% Confidence Interval** | **p-value** |
| --- | --- | --- | --- |
| Omitting long (>10 Mb) ROHs | -3.87 | [-13.60, 5.85] | 0.43 |
| Omitting people with long ROHs | -2.94 | [-8.69, 2.80] | 0.32 |
| Not controlling for missingness | -0.15 | [-4.83, 4.53] | 0.95 |
| Not controlling for average heterozygosity | 0.58 | [-2.74, 3.90] | 0.73 |
| Controlling for 10 PCs | 0.31 | [-4.37, 4.99] | 0.9 |
| Including only the longest ROH for individuals | -11.82 | [-29.23, 5.58] | 0.18 |
