# Supplementary figures and images for: No Reliable Association between Runs of Homozygosity and Schizophrenia in a Well-Powered Replication Study
Source: PLoS Genet. 2016 Oct 28;12(10):e1006343. doi: 10.1371/journal.pgen.1006343 (PMC5085024; doi:10.1371/journal.pgen.1006343)

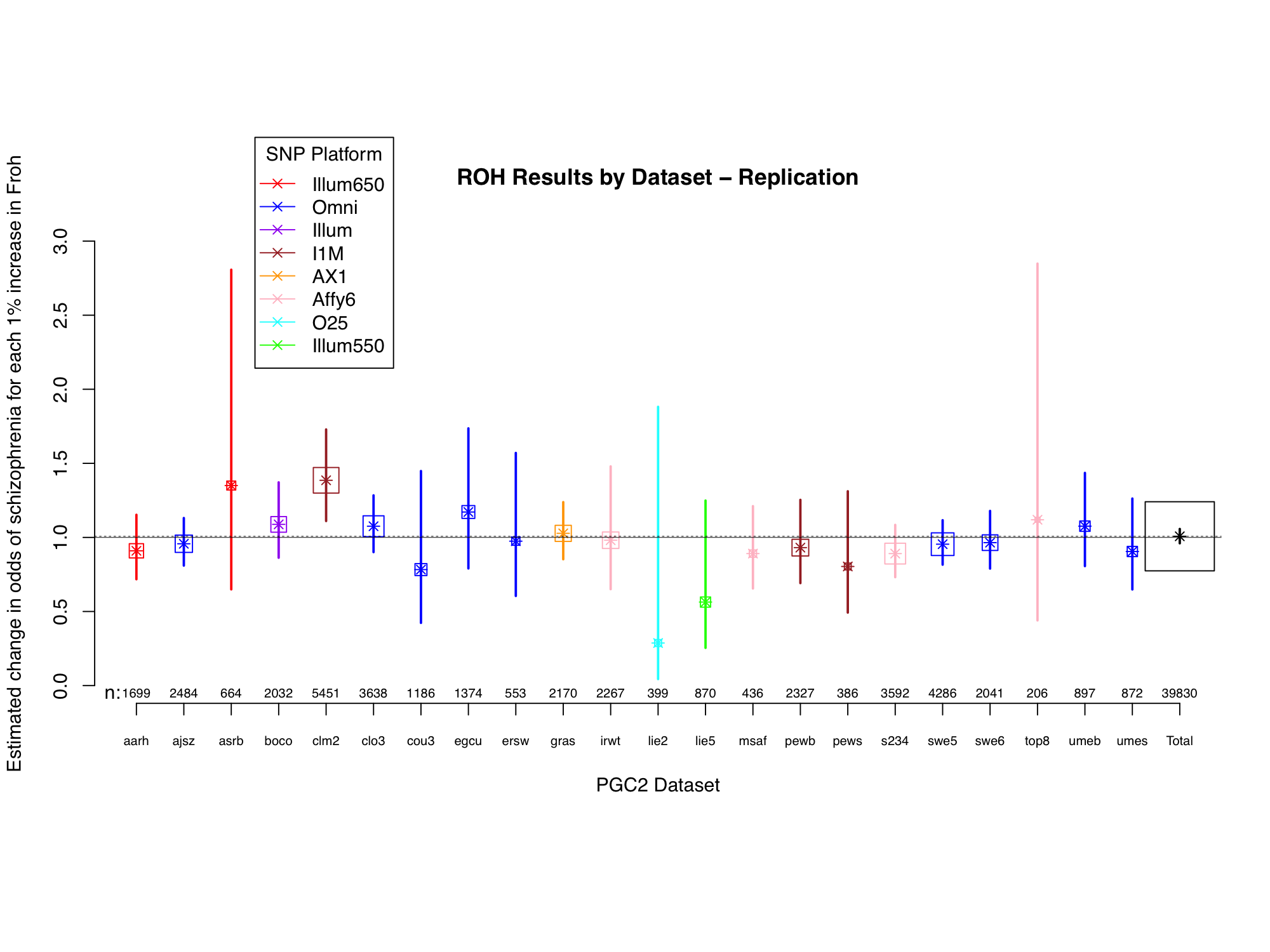

Supplement: S1 Fig — Boxes are proportional to the square root of sample sizes (also shown at the bottom). Dataset names are on the x-axis. Only one of the individual estimated odds ratios significantly differs from one (“clm2” dataset), and the overall effect (black) is not significant (β = 0.75, Z = 0.31, p = 0.76). (TIFF) [file pgen.1006343.s003.tiff]

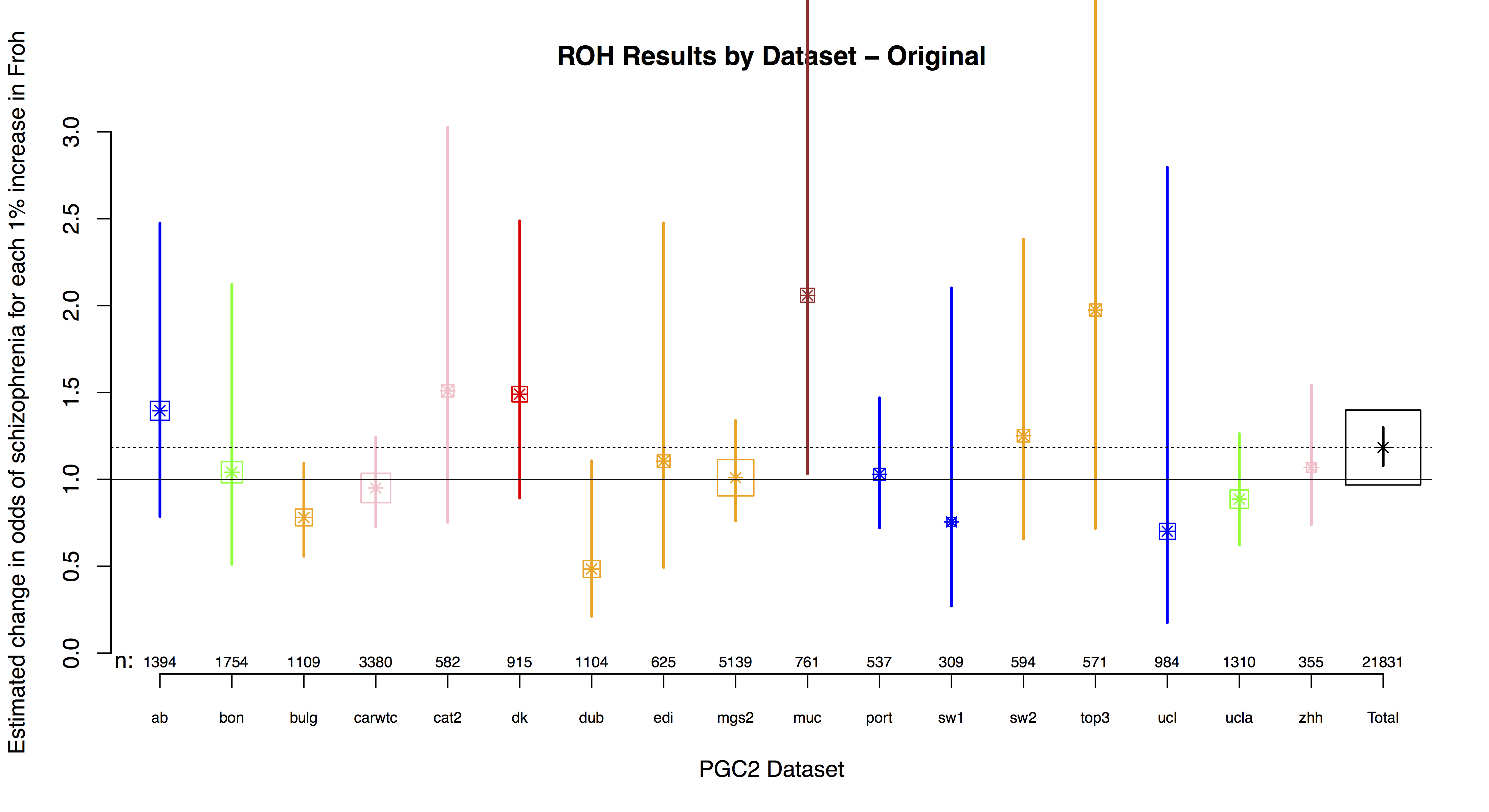

Supplement: S2 Fig — Boxes are proportional to the square root of sample sizes (also shown at the bottom). Dataset names are on the x-axis. (While the y-axis is cut off at 3 for clarity, it should be noted that the upper limit of the 95% confidence interval is 4.1 for the “muc” dataset and 5.4 for the “top3” dataset.) Only one of the individual estimated odds ratios significantly differ from one (the “muc” dataset), but the overall effect (black) is significant (Beta = 16.83, p = 0.000357.) (TIFF) [file pgen.1006343.s004.tiff]

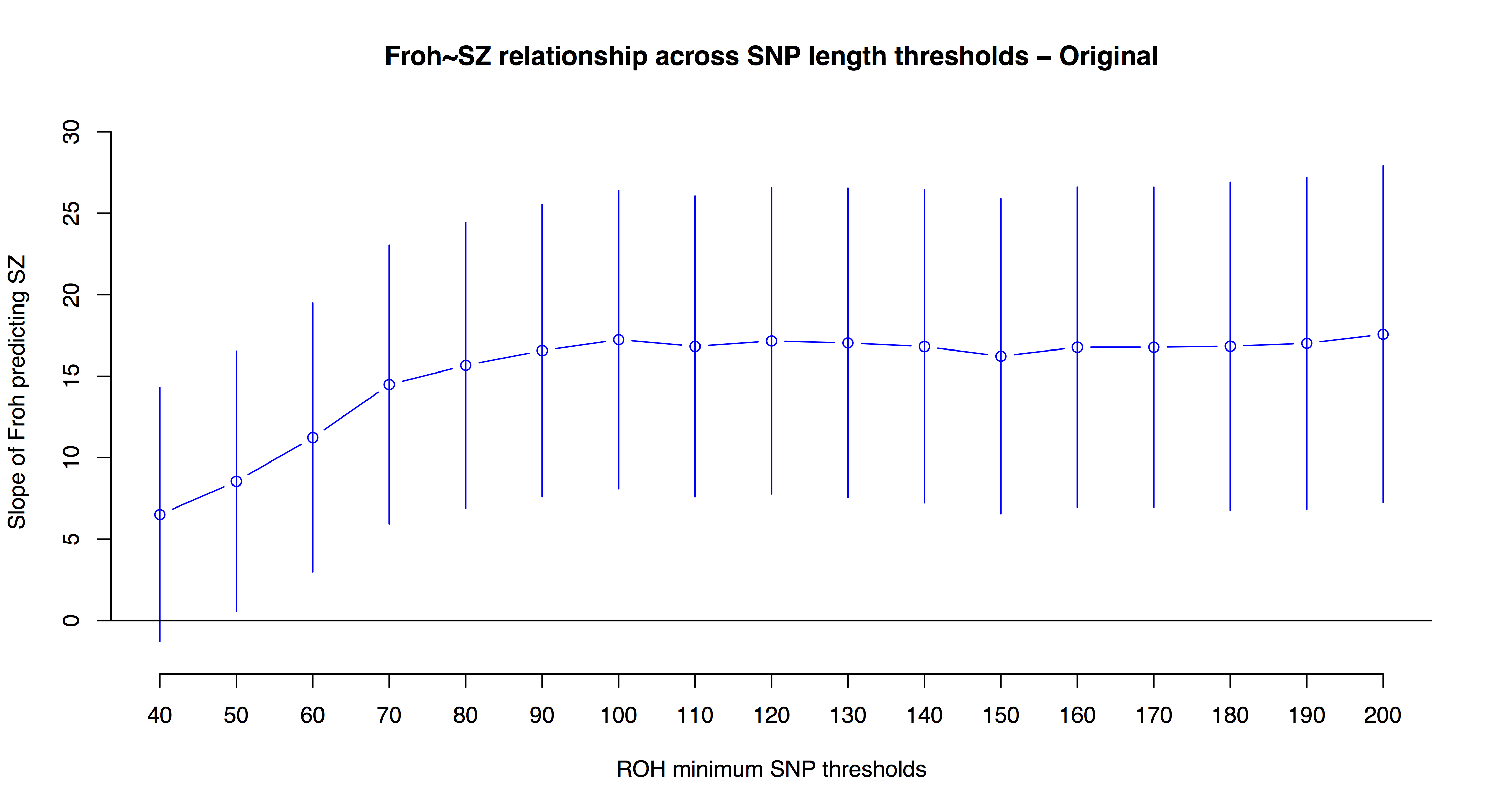

Supplement: S3 Fig — All SNP homozygosity thresholds above 40 SNPs-in-a-row were significant. (TIFF) [file pgen.1006343.s005.tiff]

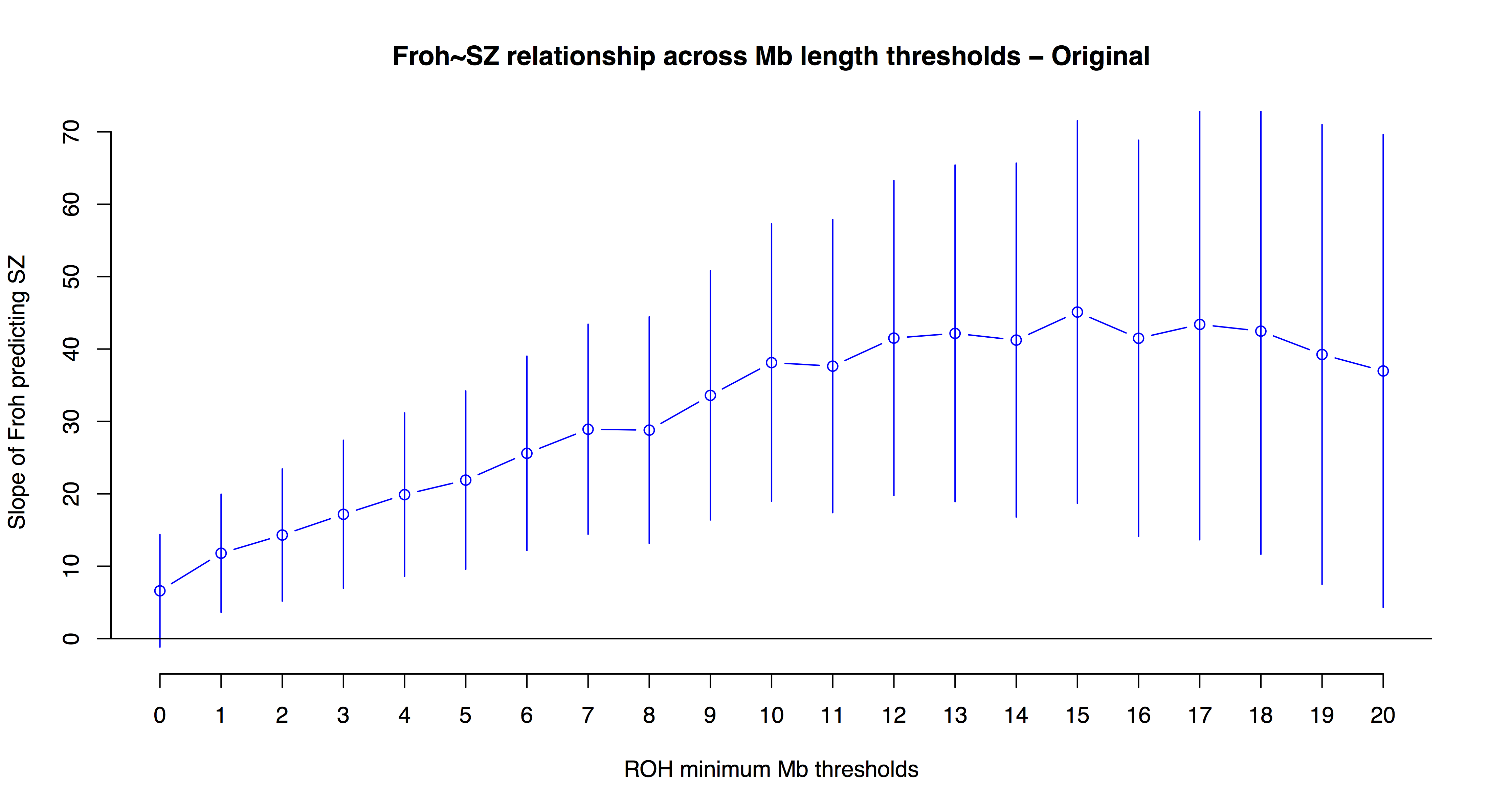

Supplement: S4 Fig — All ROH Mb thresholds equal to and longer than 1 Mb were significant. (TIFF) [file pgen.1006343.s006.tiff]

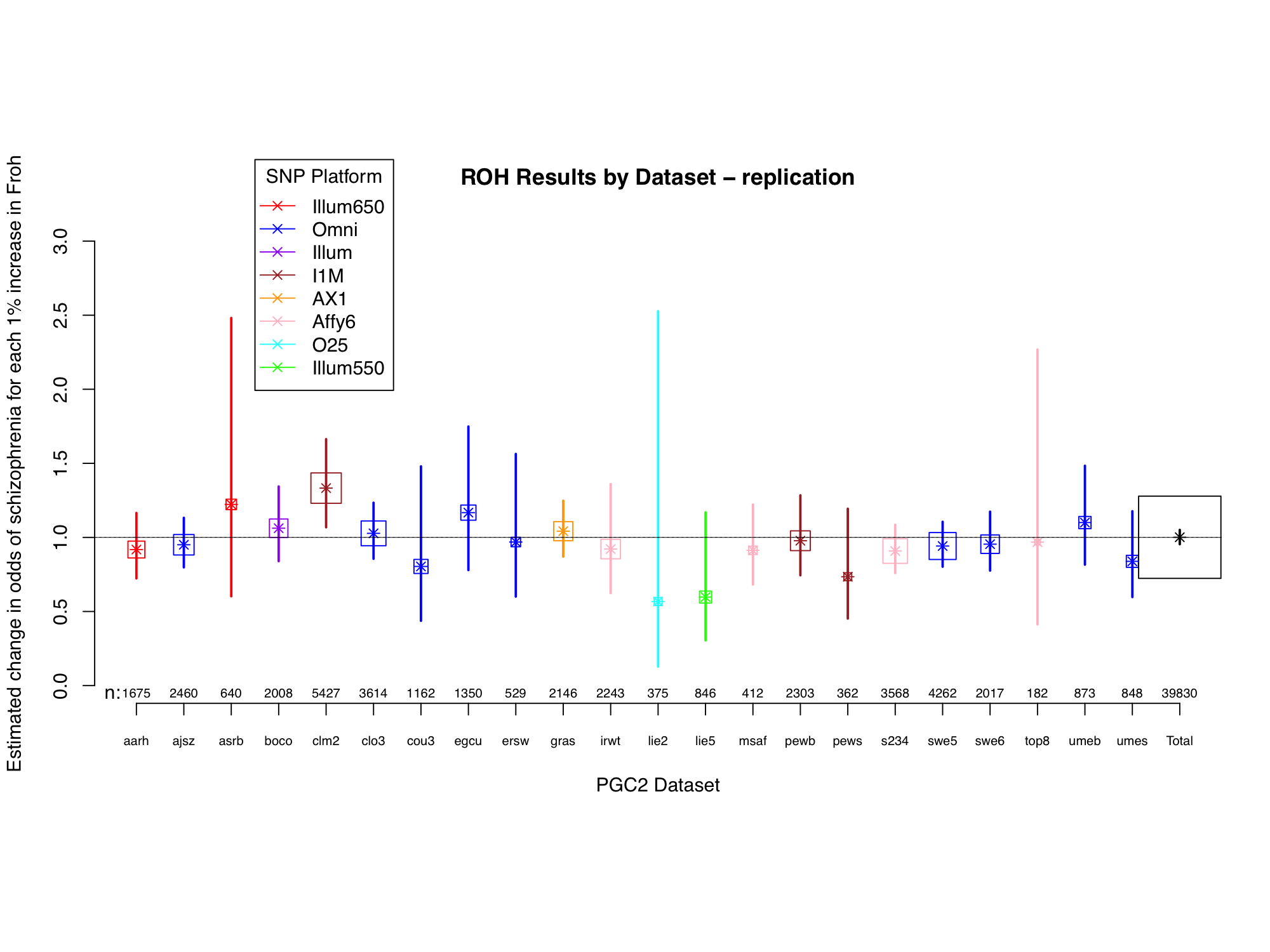

Supplement: S5 Fig — Boxes are proportional to the square root of sample sizes (also shown at the bottom). Dataset names are on the x-axis. Only one of the individual estimated odds ratios significantly differs from one (“clm2” dataset), and the overall effect (black) is not significant (β = 0.11, Z = 0.05, p = 0.96). (TIFF) [file pgen.1006343.s007.tiff]

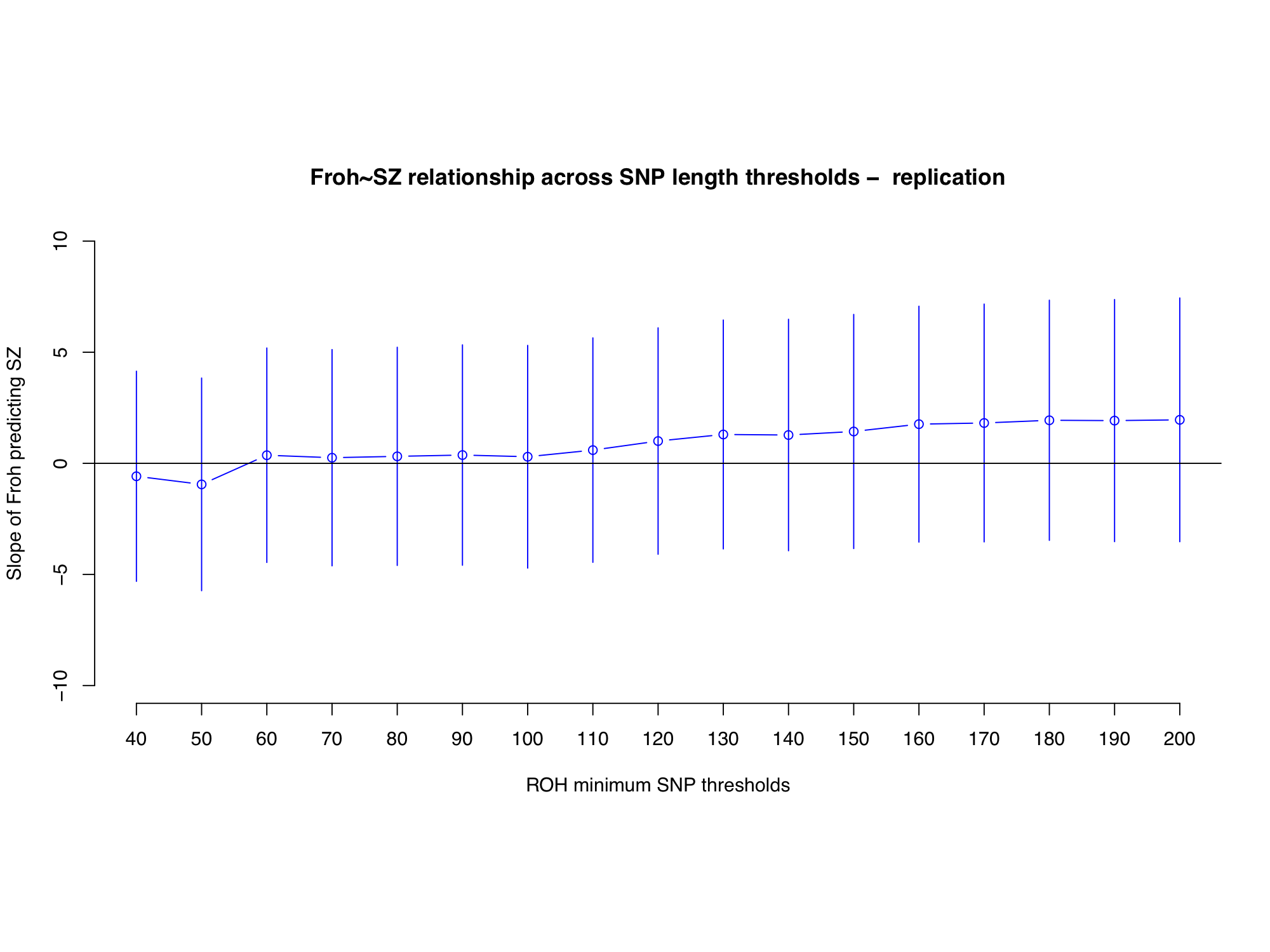

Supplement: S6 Fig — No SNP homozygosity thresholds were significant. (TIFF) [file pgen.1006343.s008.tiff]

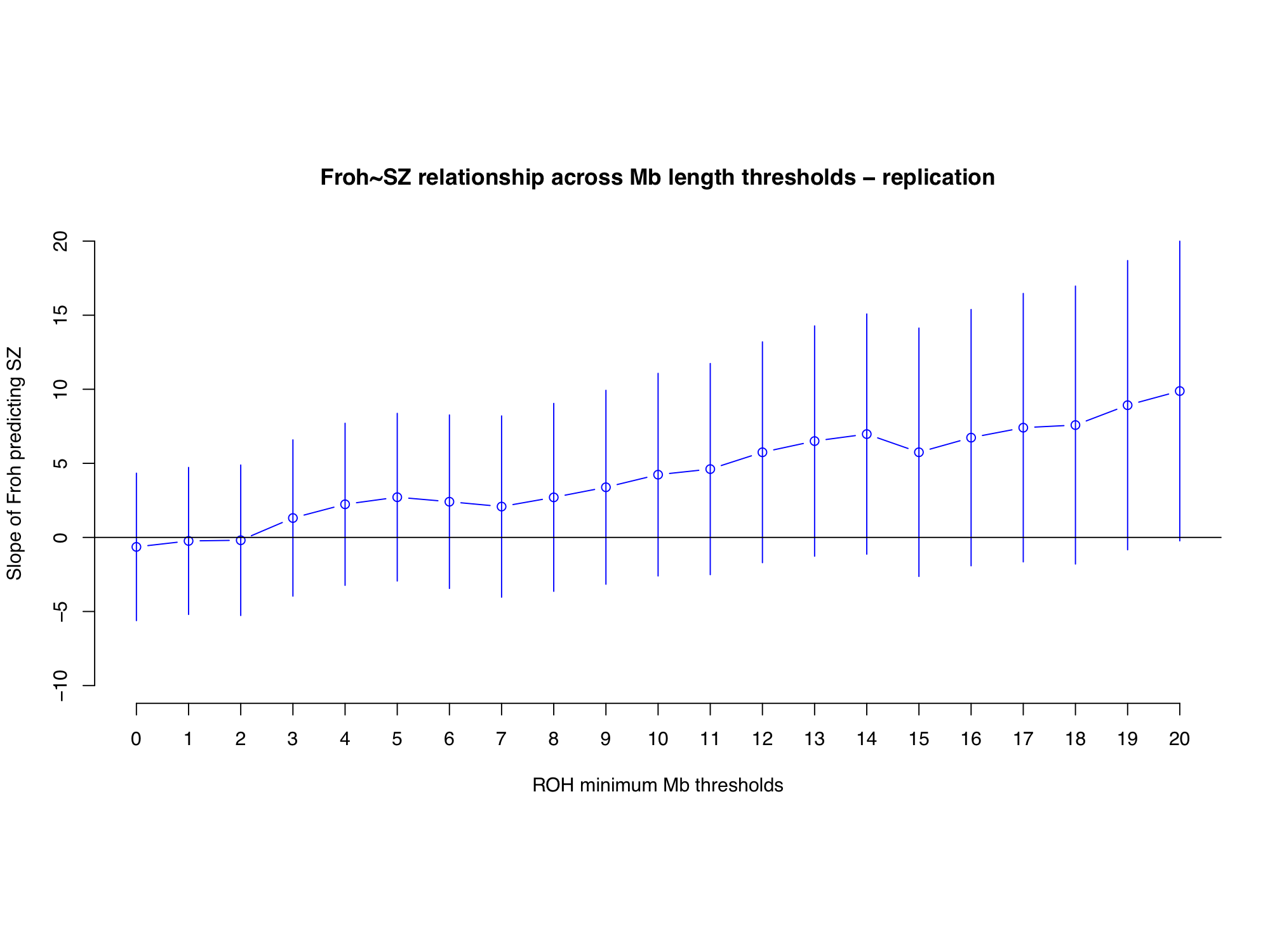

Supplement: S7 Fig — No ROH Mb thresholds were significant. (TIFF) [file pgen.1006343.s009.tiff]

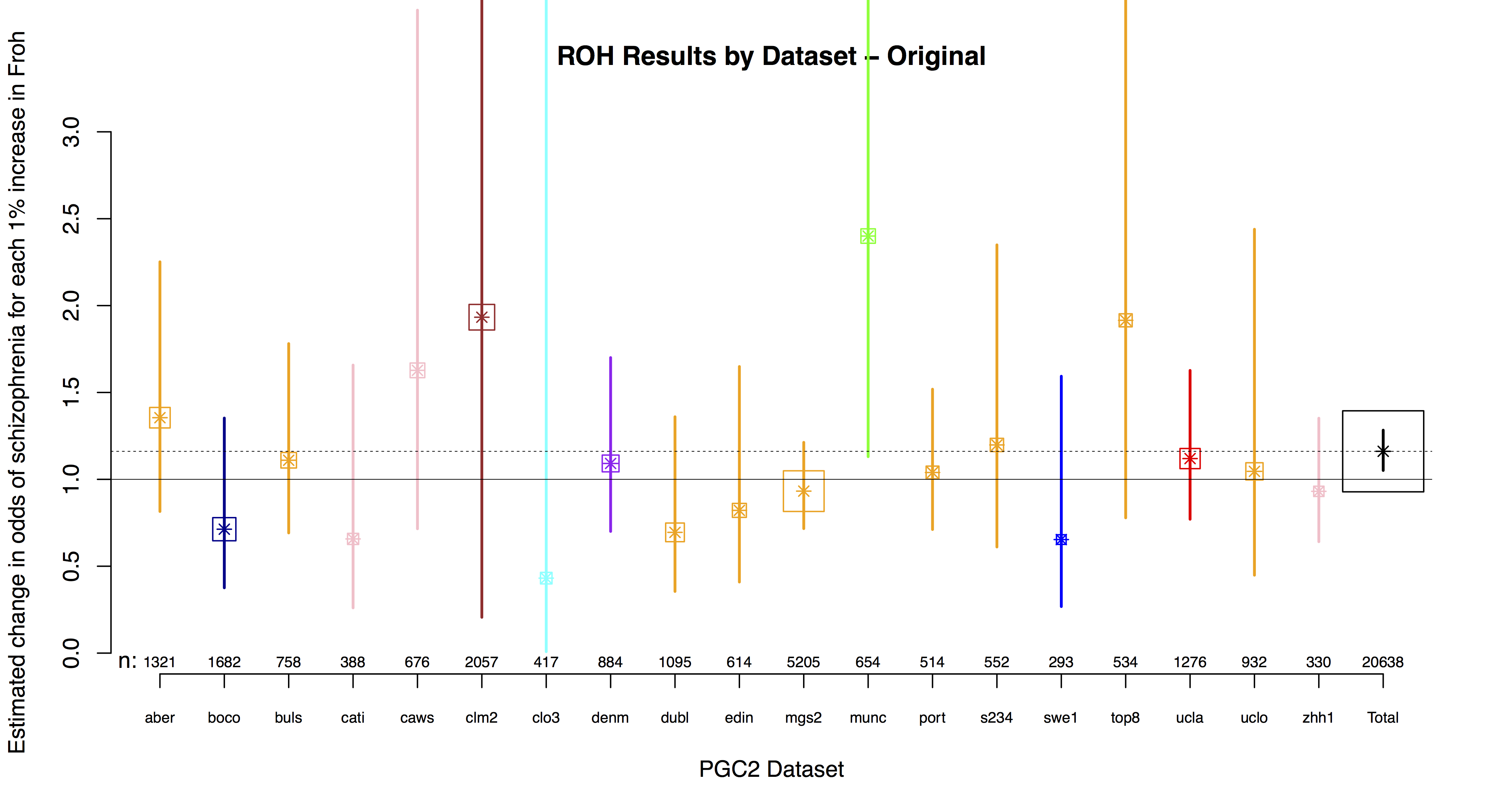

Supplement: S8 Fig — Boxes are proportional to the square root of sample sizes (also shown at the bottom). Dataset names are on the x-axis—note that this imputed analysis was performed on the original SCZ1 individuals but within the PGC’s SCZ2 data, where some of the original individuals were divided among several new datasets. This is why some of the dataset names are slightly different from those in the original unimputed PGC SCZ1 data in S2 Fig. Only one of the individual estimated odds ratios significantly differs from one, the “munc” dataset, but the overall effect (black) is significant (Beta = 14.88, Z = 2.43, p = 0.02.) (TIFF) [file pgen.1006343.s010.tiff]

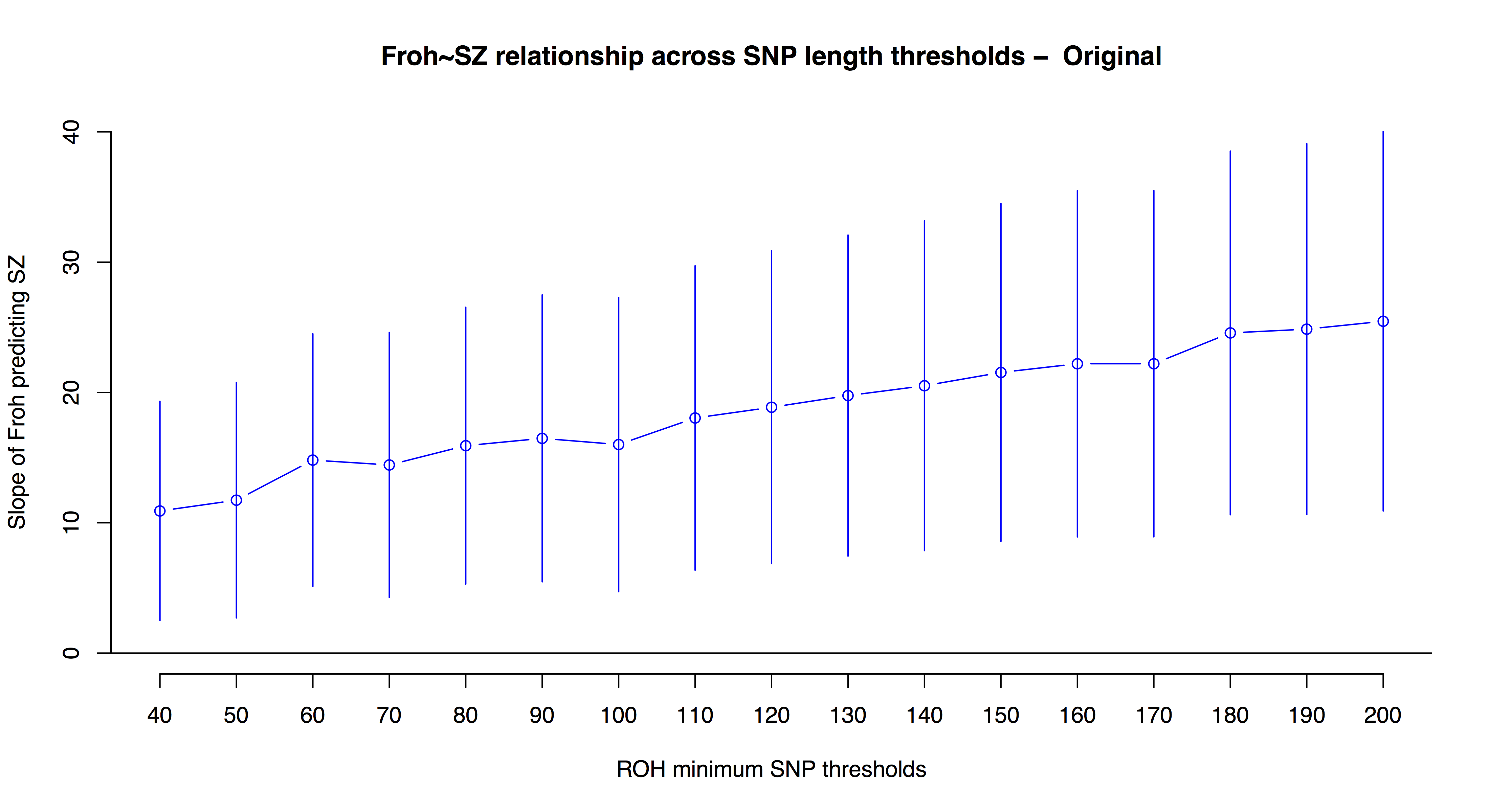

Supplement: S9 Fig — All SNP length thresholds were significant. (TIFF) [file pgen.1006343.s011.tiff]

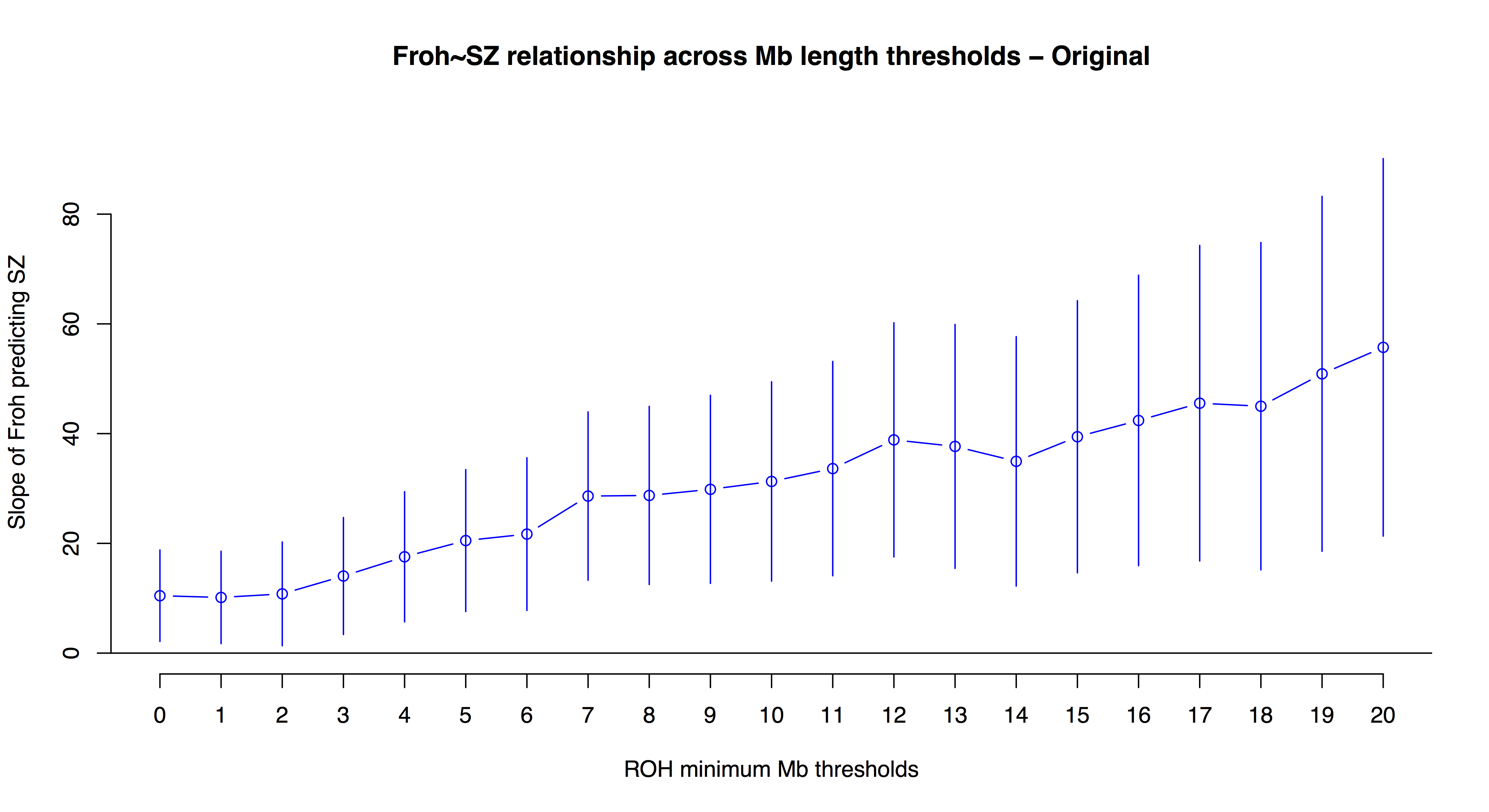

Supplement: S10 Fig — All ROH Mb thresholds were significant. (TIFF) [file pgen.1006343.s012.tiff]

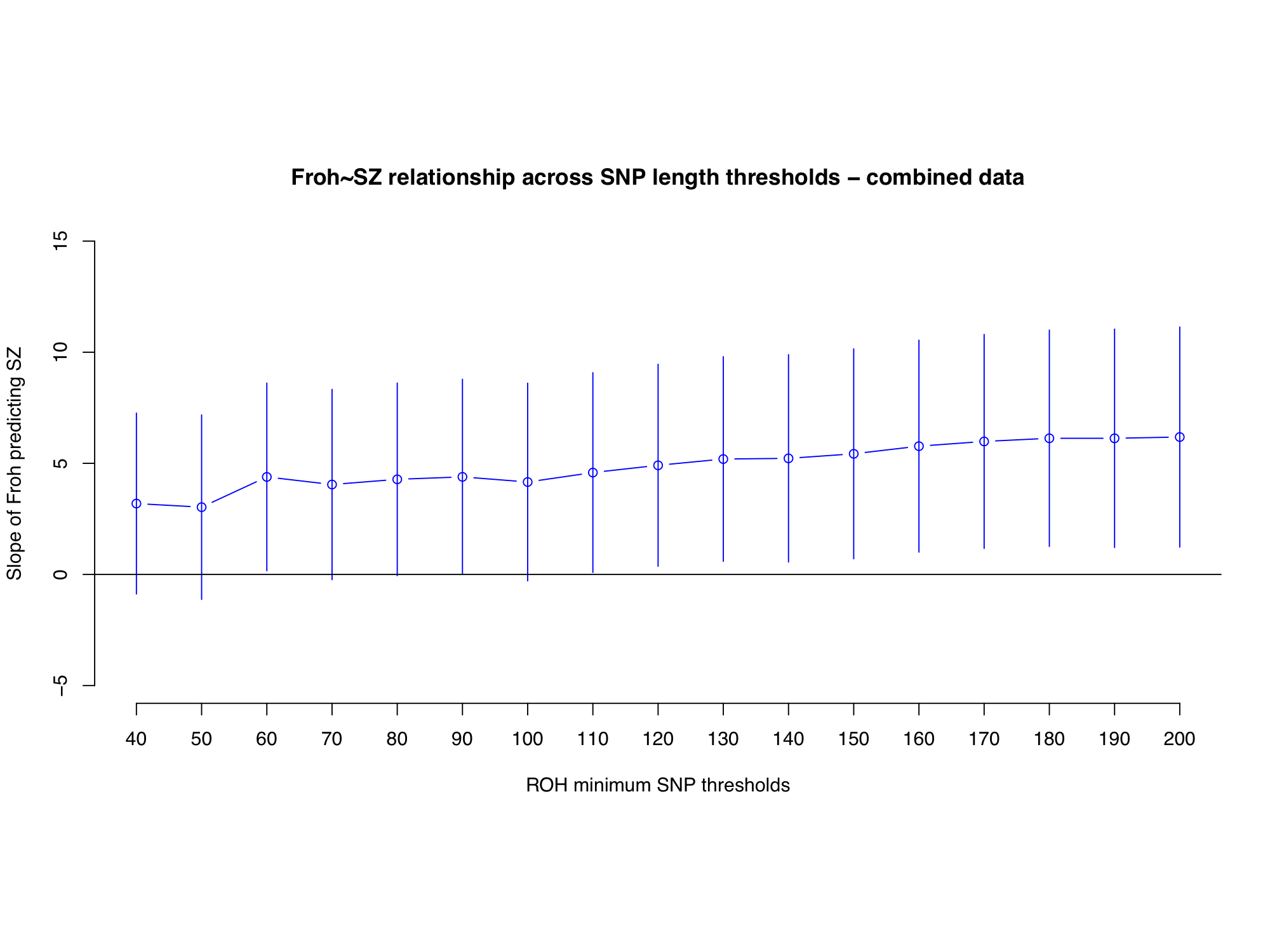

Supplement: S11 Fig — SNP thresholds of 120 homozygous SNPs-in-a-row and above were significant. (TIFF) [file pgen.1006343.s013.tiff]

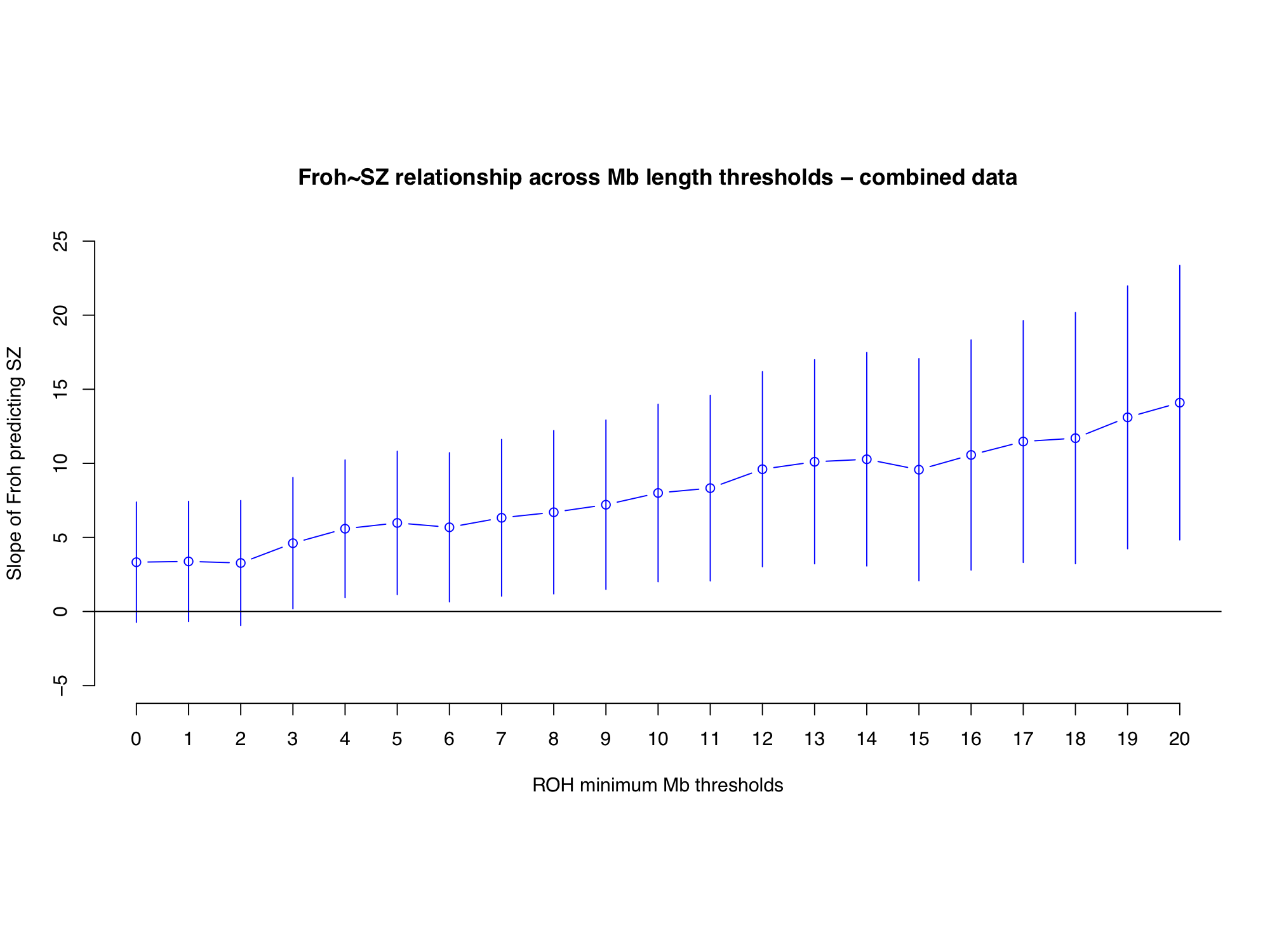

Supplement: S12 Fig — All Mb thresholds ≥ 3 Mb were significant (TIFF) [file pgen.1006343.s014.tiff]
